# MSpectraAI: a powerful platform for deciphering proteome profiling of multi-tumor mass spectrometry data by using deep neural networks

**DOI:** 10.1186/s12859-020-03783-0

**Published:** 2020-10-07

**Authors:** Shisheng Wang, Hongwen Zhu, Hu Zhou, Jingqiu Cheng, Hao Yang

**Affiliations:** 1 West China-Washington Mitochondria and Metabolism Research Center; Key Lab of Transplant Engineering and Immu-Nology, MOH, Regenerative Medicine Research Center, West China Hospital, Sichuan University, No. 88, Keyuan South Road, Hi-tech Zone, Chengdu, 610041 China; 2 Shanghai Institute of Materia Medica, Chinese Academy of Sciences, Shanghai, China

**Keywords:** Raw mass spectrometry data, Proteome profiling, Feature swath extraction, Deep neural networks, Multi-tumor types, Leave-one-out cross prediction strategy

## Abstract

**Background:**

Mass spectrometry (MS) has become a promising analytical technique to acquire proteomics information for the characterization of biological samples. Nevertheless, most studies focus on the final proteins identified through a suite of algorithms by using partial MS spectra to compare with the sequence database, while the pattern recognition and classification of raw mass-spectrometric data remain unresolved.

**Results:**

We developed an open-source and comprehensive platform, named MSpectraAI, for analyzing large-scale MS data through deep neural networks (DNNs); this system involves spectral-feature swath extraction, classification, and visualization. Moreover, this platform allows users to create their own DNN model by using Keras. To evaluate this tool, we collected the publicly available proteomics datasets of six tumor types (a total of 7,997,805 mass spectra) from the ProteomeXchange consortium and classified the samples based on the spectra profiling. The results suggest that MSpectraAI can distinguish different types of samples based on the fingerprint spectrum and achieve better prediction accuracy in MS1 level (average 0.967).

**Conclusion:**

This study deciphers proteome profiling of raw mass spectrometry data and broadens the promising application of the classification and prediction of proteomics data from multi-tumor samples using deep learning methods. MSpectraAI also shows a better performance compared to the other classical machine learning approaches.

## Background

The comparison of molecular features from diverse physiological or disease states is vital for determining different potential biomarkers closely associated with specific diseases [1, 2]. For example, identification of cancer subtype-specific biomarkers and candidate drivers can reveal useful insights into disease pathogenesis and facilitate personalized cancer therapy [3]. Fortunately, proteomics can provide a heuristic scheme for this purpose. Over the past decades, liquid chromatography coupled with mass spectrometry (LC–MS) has enabled the high-throughput analysis of intact proteins or peptides from trypsinized protein mixtures in complex samples according to their specific retention time and mass-to-charge value (*m/z* value), which provides great spectral data information for proteome analysis [4–6]. Thus, this approach can help in analyzing large-scale biological samples and has progressively become the prevalent and core technique of choice for global and unbiased characterization of proteome alterations in various sample conditions. However, most studies have focused on identifying and quantifying proteins through algorithm-directed sequential database searching by using MS spectral data [7–9]. Little information exists about the contribution of the original mass spectrometry data to sample classification before the data can be decoded into the corresponding peptides and proteins. Therefore, there is an urgent need of developing highly efficient data-processing methods to extract and analyze the large-scale and multidimensional raw spectrum data, especially generated from clinical samples.

A number of approaches and tools based on versatile algorithms have been developed, including typical machine learning approaches, such as logistic regression [10], kNN algorithm [11], support vector machine (SVM) [12], and decision-tree algorithm [13]. When running these algorithms, data preprocessing, such as feature extraction or selection, is a recommendatory step for sample classification [14]. However, the effect of feature extraction and prediction accuracy are not invariably satisfactory when using these conventional machine-learning methods for high-dimensional data. In contrast, deep learning, which processes the application of multilayered artificial neural networks (ANNs) to learning tasks [15], can discover useful features independently, thus eliminating biases proposed by manual engineered features [16]. Deep learning methods, such as convolutional neural networks (CNNs) and recurrent neural networks (RNNs), have been repeatedly proved to outperform the aforementioned state-of-the-art classical machine-learning algorithms for high-dimensional data [17].

In this study, we present an open-source and powerful platform, MSpectraAI (Mass Spectra Artificial Intelligence), as an easy-to-use stand-alone software for practical extraction and analysis of large-scale and multidimensional raw mass-spectrometric data with deep neural networks (DNNs), which is a type of deep learning (and a complex neural network-based) model [18]. To date, this platform contains (1) feature swath extraction, in which all collected mass spectra are acquired consistently with sequential windows; (2) sample classification, in which different group samples can be tested and predicted using an ANNs model; (3) visualization, in which the fingerprint of mass spectra and model prediction results are shown as vector graphs. Moreover, this platform provides downloadable tabular data results in the csv format for further user-based analysis. MSpectraAI can be processed locally and handled easily by users, even without any bioinformatics background, to analyze complicated data, especially obtained from clinical samples. Expansively, professional users can also design their own DNN model and run it in this tool. For demonstrating the originality and application of this software, six tumor types, with a total of 7,997,805 mass spectra, were downloaded and assembled from the ProteomeXchange consortium [19]. Further analysis reveals the existence of the diversity of mass spectra profiling in different types of samples; MSpectraAI can make a prediction to classify these complex clinical samples based on their spectra profiling in each tumor type. In view of this, MSpectraAI shows promising potential for the practical application of clinical settings in the precision medicine era.

## Implementation

### Dependencies

All functions in MSpectraAI were written in R (https://www.r-project.org/) [20], and the graphical user interface (GUI) was developed in Shiny (https://github.com/rstudio/shiny). Therefore, R and relative packages are supposed to be installed in advance if users decide to operate this tool locally. Particularly, the DNN model was built using Keras (version 2.2.4) (https://github.com/fchollet/keras), which must also be preconfigured on the system. The detailed installation manual can be found in the Additional file 1. MSpectraAI is an open-source platform available on the GitHub repository, https://github.com/wangshisheng/MSpectraAI.

Additionally, MSpectraAI can also be run locally on Windows, Linux, and Mac operating systems. It does not require any specific hardware configuration; however, performances are dependent on the amount of available computer memory and the number of CPU cores or GPU settings (NVIDIA Quadro K2200). Specially, MSpectraAI supports professional users to compile their own DNN methods, and even more complicated deep learning models, to process large dimensional data.

### Study design and analysis workflow

**Fig. 1:**
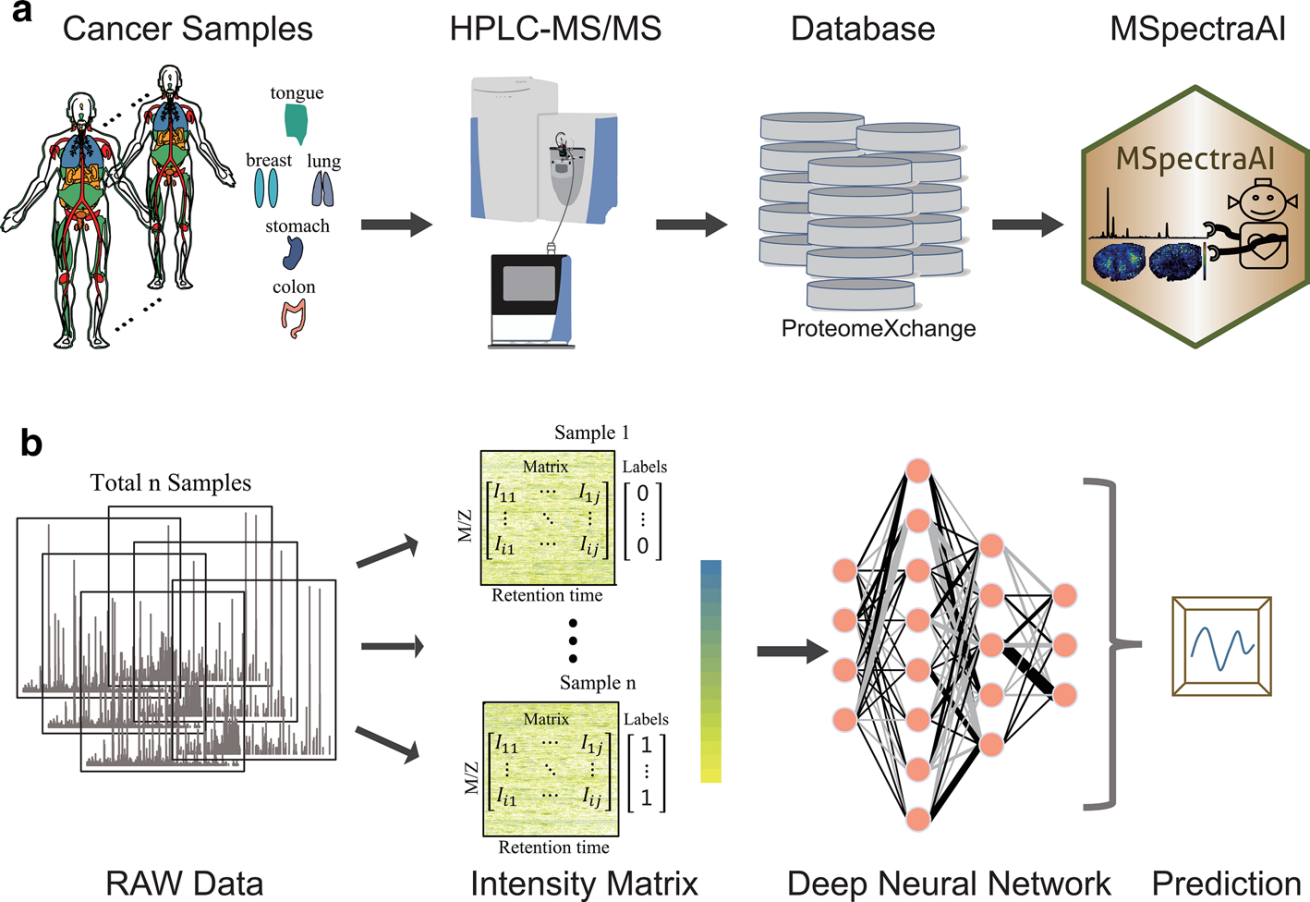
Overall pipeline of MSpectraAI. **a** Data of the six tumor types were obtained based on the HPLC–MS/MS method, downloaded from Pro-teomeXchange database, and then imported into MSpectraAI. **b** Skeleton diagram of data analysis in MSpectraAI

**Fig. 2:**
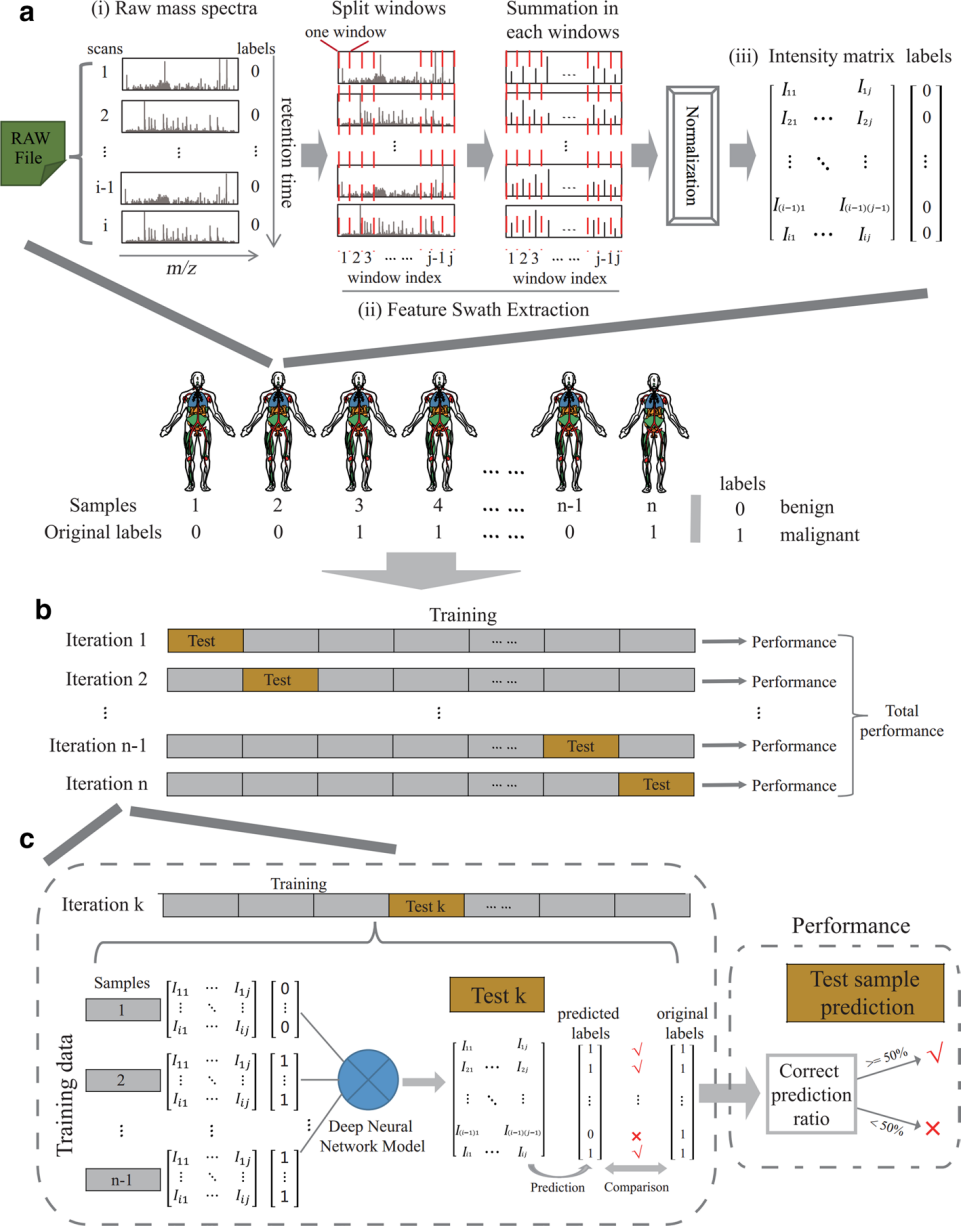
Detailed implementation of data process logic in MSpectraAI. **a** Workflow of data transformation from raw file to intensity matrix. Graphs show an example of one sample with original label 0. (i) Raw mass spectra. There are total i scans in this raw file and the original label of each scan is marked with 0 (As the sample is labeled 0). (ii) Feature Swath Extraction. Split windows across *m/z* dimension (The range between two red dashed lines is referred to as one “window”, j means total window number) and sum all peak intensities in each window (formula (1)). (iii) Intensity matrix. After summation, the intensities in each scan are normalized by dividing the maximum intensity of each scan (formula (2)). Finally, we obtain the intensity matrix and corresponding label matrix. **b** Leave-one-out cross prediction strategy. In each independent iteration, one single sample as the independent test data set (gold color), and the remaining samples as the training data (grey color). Then we can estimate total performance based on every iteration result. **c** The computational framework in each iteration. Graphs show an example of kth iteration (the kth sample with original label 1 as test data). In the left dashed box, the deep neural network model is trained by training data sets, then we predict each row (scan) in test data using this model. The predicted labels can be compared with original labels, if same, marked with “√”, otherwise, “×”. In the right dashed box, if more than 50% of the scans in the test sample are predicted correctly, this means the pre-diction for test sample is correct (“√”), otherwise, wrong (“×”)

The overall pipeline of MSpectraAI is shown in Fig. 1. To validate the performance of MSpectraAI, we further tested the platform on datasets of six tumor types (Table 1; Additional file 1: Table S1) from ProteomeXchange consortium (Fig. 1a), which is one of the world-leading data repositories of MS-based proteomics data [19]. All data were captured using the data-dependent-acquisition (DDA) method [21] and the corresponding LC–MS/MS parameters were summarized in the Additional file 1: Table S2. In total, there are 7,997,805 raw mass spectra, including 1,349,180 parent ions mass spectra (MS1 scan) and 6,648,625 daughter ions mass spectra (MS2 scan). Next, all original data (.raw/.wiff/.RAW files) need to be converted into mzXML or mzML format (Additional file 1: Fig. S1) by using the RawConverter software [22]; optionally, users can also choose other similar software, such as MSConvert [23]. These raw data were then orderly transformed into regular intensity matrices for the subsequent DNN model (Fig. 1b) to perform samples classification/prediction by using a homemade approach named feature swath extraction (Fig. 2a), which is inspired by the data-independent-acquisition (DIA) method [24].

**Table 1:** Sample information of six tumor types

Names	PXD IDs	Raw file number	Spectra number (MS1/MS2)
Oral cancer	PXD007232 [25]	10	45,440
			542,145
Breast cancer	PXD008012 [26]	50	77,685
			748,881
Head and neck lung cancer	PXD007705 [27]	32	478,767
			4,125,430
Nonsmall cell lung cancer	PXD005698 [28]	24	136,575
			788,739
Gastric cancer	PXD002213 [29]	34	558,795
			261,150
Colorectal cancer	PXD009602 [30]	20	51,918
			182,280
SUM	6	170	7,997,805

### Core algorithm implementation

**Fig. 3:**
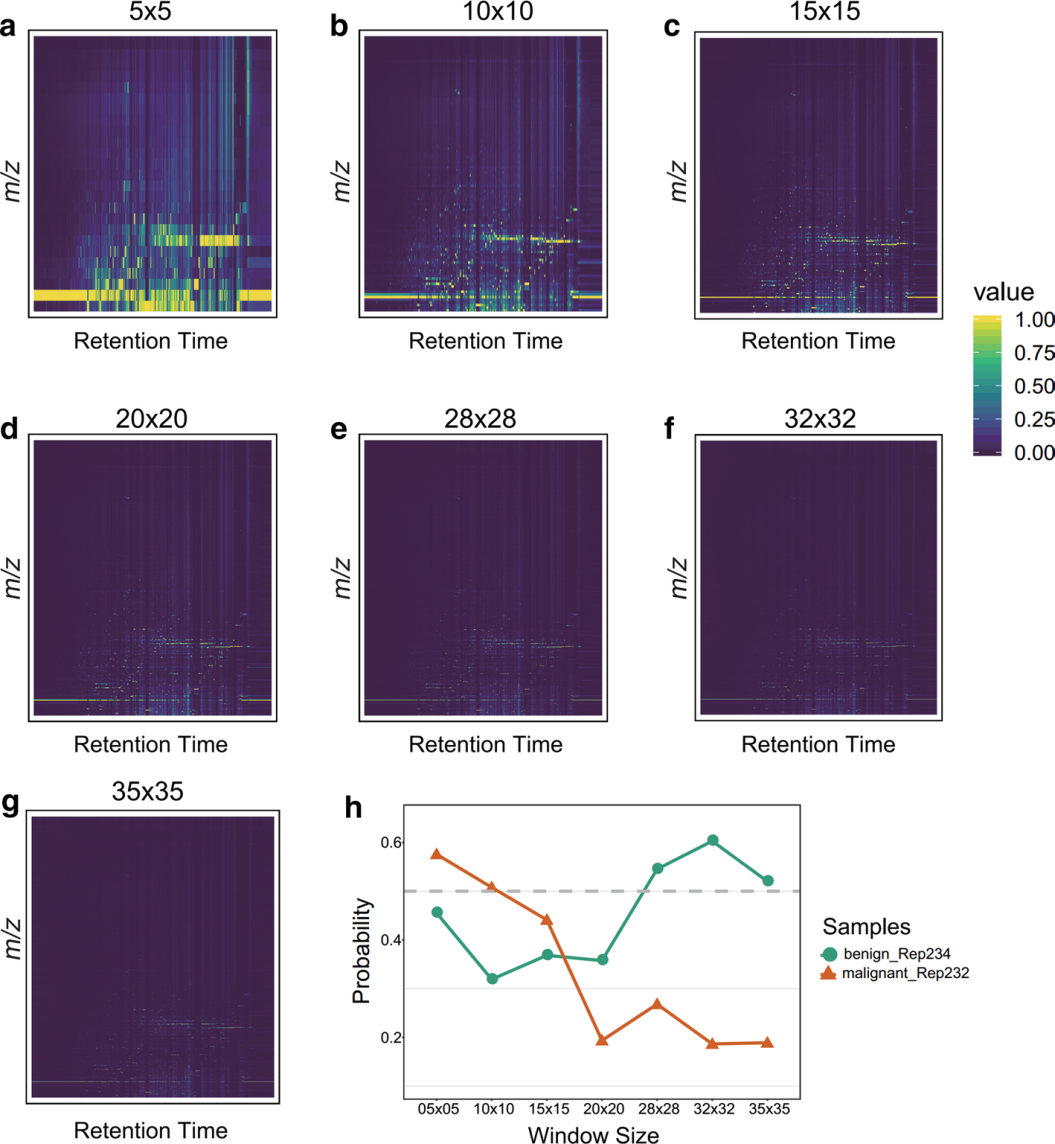
**a**–**g** Seven window sizes were used for the colorectal malignant samples to determine the peak-intensity distribution. **h** The trend of pre-diction accuracy with the decreasing window size (the grey dashed line means 0.5 probability). ‘5 × 5′ means that there are total 25 windows across the *m/z* dimension, ‘10 × 10′ means there are total 100 windows, and so on. The color here from deep viridis to deep yellow means the intensity values change from low to large

The fundamental data process logistic flowchart of MSpectraAI is illustrated in Fig. 2, which contains two main parts:

1. *Feature Swath Extraction* (Fig. 2a). This step is mainly to obtain the normalized intensity matrix and the label matrix. In most situations, the range of ion *m/z* scanning and number of peaks in each spectrum dynamically change; this is not suitable for analysis using a deep learning model. Therefore, these data should first be structured uniformly. Here, we firstly divide the whole *m/z* range into equal windows. The window size here can be designed freely by users according to the complexity of their data (Detailed in Additional file 1: Notes 9.4) and they can take our results (Fig. 3) as references. Then all peaks within the same window are summed together across the *m/z* dimension in each mass spectrum:

1 $$IM = \left[ {\begin{array}{*{20}c} {I_{11} } & \cdots & {I_{1j} } \\ \vdots & \ddots & \vdots \\ {I_{i1} } & \cdots & {I_{ij} } \\ \end{array} } \right] = \left[ {\begin{array}{*{20}c} {\mathop \sum \limits_{k1}^{n1} p_{k11} } & \cdots & {\mathop \sum \limits_{kj}^{nj} p_{k1j} } \\ \vdots & \ddots & \vdots \\ {\mathop \sum \limits_{ki}^{ni} p_{ki1} } & \cdots & {\mathop \sum \limits_{kij}^{nij} p_{kij} } \\ \end{array} } \right]$$

where *IM* implies intensity matrix, *i* denotes the MS scan index, *j* denotes the window index, and $\mathop \sum \limits_{kij}^{nij} p_{kij}$ denotes the summation of all peaks within the *i*th scan and *j*th window. As the scale of peak intensities in each window are inconsonant, moreover, the intensities need to be normalized by dividing the maximum intensity of each scan:

2 $$NIM = {\raise0.7ex\hbox{${IM}$} \!\mathord{\left/ {\vphantom {{IM} {(\max (I_{1,1 \ldots j} ), \ldots , \max (I_{i,1 \ldots j} ))}}}\right.\kern-\nulldelimiterspace} \!\lower0.7ex\hbox{${(\max (I_{1,1 \ldots j} ), \ldots , \max (I_{i,1 \ldots j} ))}$}}$$

where *NIM* means normalized intensity matrix, *i* denotes the MS scan index, *j* denotes the window index. In addition, the label matrix is designed based on the sample classes, for instance, if the normal samples are marked with 0, its original label matrix is [0, 0, …, 0], similarly, the tumor samples are marked with 1, its original label matrix can be [1, 1, …, 1]. The label matrix length are equal to scan number in the corresponding sample.

2. *Leave-one-out cross prediction strategy* (Fig. 2b, c). For each tumor type, by default, leave-one-out cross prediction strategy was implemented for data analysis [31], in which one single observation from the original samples as the independent test data set, and the remaining observations as the training data in each for loop (Fig. 2b). Optionally, users can also regulate the data allocation in the training and testing processes by editing this software to be more suitable for their own samples. In each iteration, there are two main procedures: 1. Predicting every scan in test data (the left dashed box in Fig. 2c). We firstly design a three-layers DNN model with total 59,779 parameters (Additional file 1: Fig. S2) and train it using training data, then predict each scan in the independent test data. 2. Evaluating the prediction performance of test sample. We can compare the predicted label with the original label of each scan in test data and count the correct prediction ratio. If this ratio is equal or greater than 0.5, we here think this test sample is predicted correctly, otherwise wrong.

### Model performance, evaluation and comparison

To assess the agreement between actual sample and predicted labels of the mass spectra, we assessed the precision and recall as follows:

3 $$Precision = \frac{TP}{{\left( {TP + FP} \right)}},$$

4 $$Recall = \frac{TP}{{\left( {TP + FN} \right)}} ,$$

where true positives (TP) are the proportion for which the predicted labels match the prior tumor labels; false positives (FP) are the predicted labels that the normal has been identified incorrectly; and false negatives (FN) are labels that the tumor has been identified benign. Notably, if users define the benign samples as positive labels, the corresponding calculation should be adjusted based on the actual situation. Finally, to measure per-class performance, we calculated the F1 score, which is the weighted mean between precision and recall.

5 $$F1 = 2*\frac{Precision*Recall}{{\left( {Precision + Recall} \right)}} .$$

In addition, The ROCR package [32] was used for plotting the receiver operating characteristic (ROC) curves and calculating the area under the curve (AUC) for both MS1 and MS2 spectra data in each tumor type. Finally, we defined the accuracy for all samples of one type tumor as:

6 $$Accuracy = \frac{{N_{predicted} }}{{N_{total} }},$$

where N_predicted denotes the sum of samples in which more than half of the mass spectra are predicted correctly and N_total denotes total number of samples.

Finally, we compared four common criteria (Accuracy, Sensitivity, Precision, and F1 score) from MSpectraAI with those from the published results [25] using different types of common machine learning algorithms (Linear SVM, RBF SVM, Logistic Regression, Random Forest) and the results obtained from the classic approach utilizing MaxQuant [7] coupled with DNN model (‘MaxQuant + DNN’ mode, which means the protein matrix data were generated first with MaxQuant software and then processed by using a similar DNN model as implemented in MSpectraAI, detailed in Additional file 1: Methods), to demonstrate the classification and prediction capability of MSpectraAI.

## Results

### Window-size dynamic selection

To construct suitable data for the DNN model, the raw intensities were binned using the feature swath extraction approach (Fig. 2a). However, the peaks in different window sizes exhibit various distributions, which may provide varying degrees of information for the following deep learning model. In this study, we used seven window sizes on the colorectal malignant samples to extract the peak intensities (Fig. 3a–g). With the increasing window size, the intensity distribution became more exquisite across the *m/z* dimension. Subsequently, we predicted the same benign and malignant samples in each window size, and the remaining data with the same treatment were used in training (detailed methods in Experimental Procedures). The result (Fig. 3h) shows that the prediction accuracy improves with the appropriate decrease in window size, indicating that 28 × 28 (total 784 windows) or 32 × 32 (total 1024 windows) can be more proper for this tumor data and demonstrates that too small or too large window sizes are not conducive to examine the difference between normal and cancer samples.

### Pattern detection

**Fig. 4:**
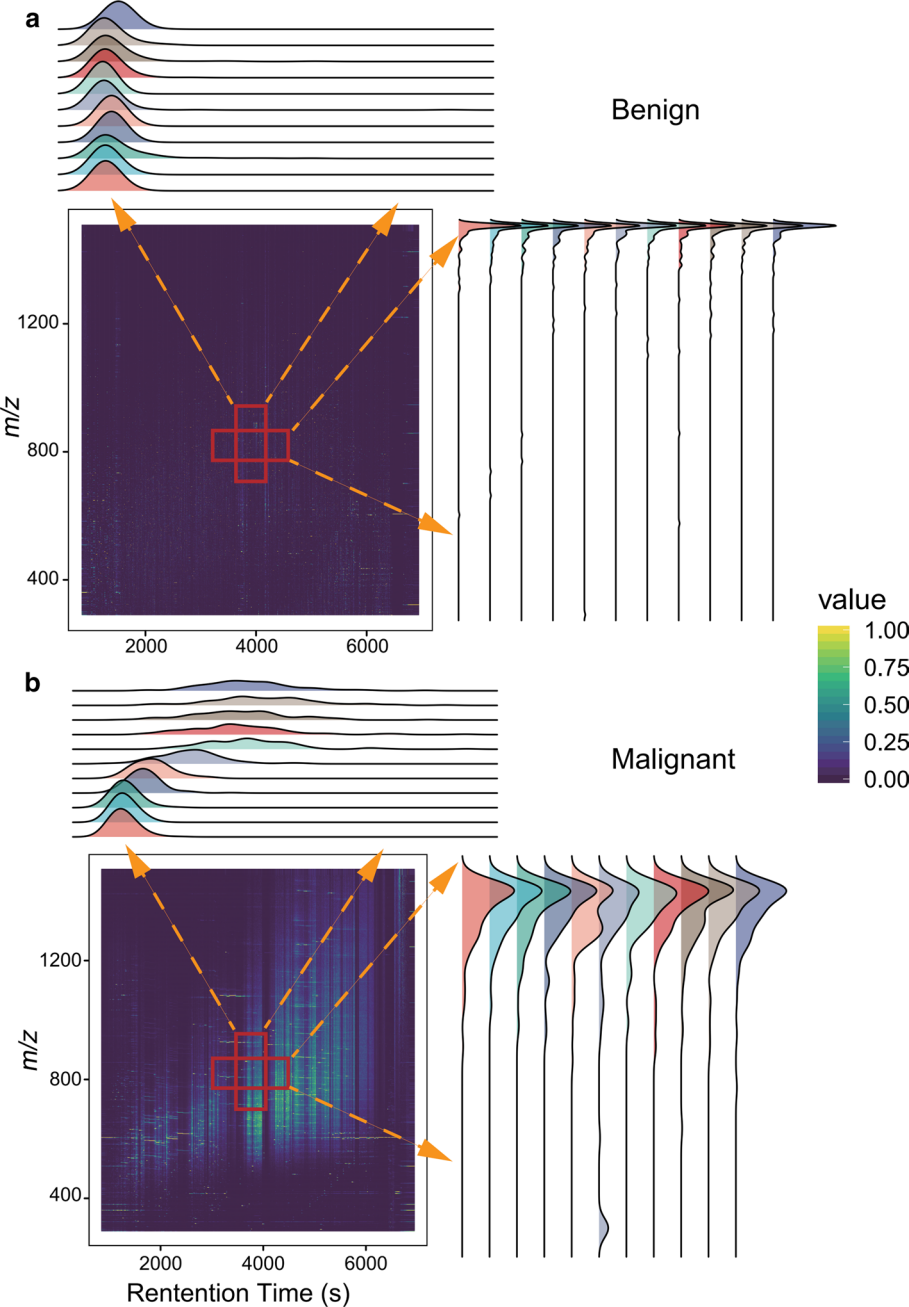
Pattern detection for **a** benign sample and **b** colorectal cancer sample across either *m/z* dimension or retention time. The heatmaps show the distribution of the intensities calculated from formula (1), where the color from deep viridis to deep yellow means the intensity values change from low to large. The density plots show the distribution of each channel intensities (e.g. the right density plots show the intensity distribution of 790–810 across *m/z* dimension, the top density plots show the intensity distribution of 3990–4010 across retention time dimension)

Pattern detection is concerned with the automated discovery of regularities in different data through the use of computer algorithms and the use of these regularities to take actions such as data classification. We firmly believe that diverse profiling exists in normal and cancer samples. Furthermore, these patterns are highly possible to be recorded in thousands of mass spectra and display different distribution. Figure 4 illustrates the considerable distinction between the benign sample and colorectal cancer sample across either *m/z* dimension or over a retention period. Overall, heatmaps show that peak intensities in malignant samples are much larger than those in benign samples, implying that the proteome destabilizes more intensely in tumor. From the viewpoint of both the *m/z* and retention time, the probability density curves are much more consistent and orderly in benign samples, while the intensity distribution in malignant samples is scraggly and un-constant. Other tumor data have also shown similar results, which users can repeat through MSpectraAI software conveniently.

### Overall performance of MSpectraAI

**Fig. 5:**
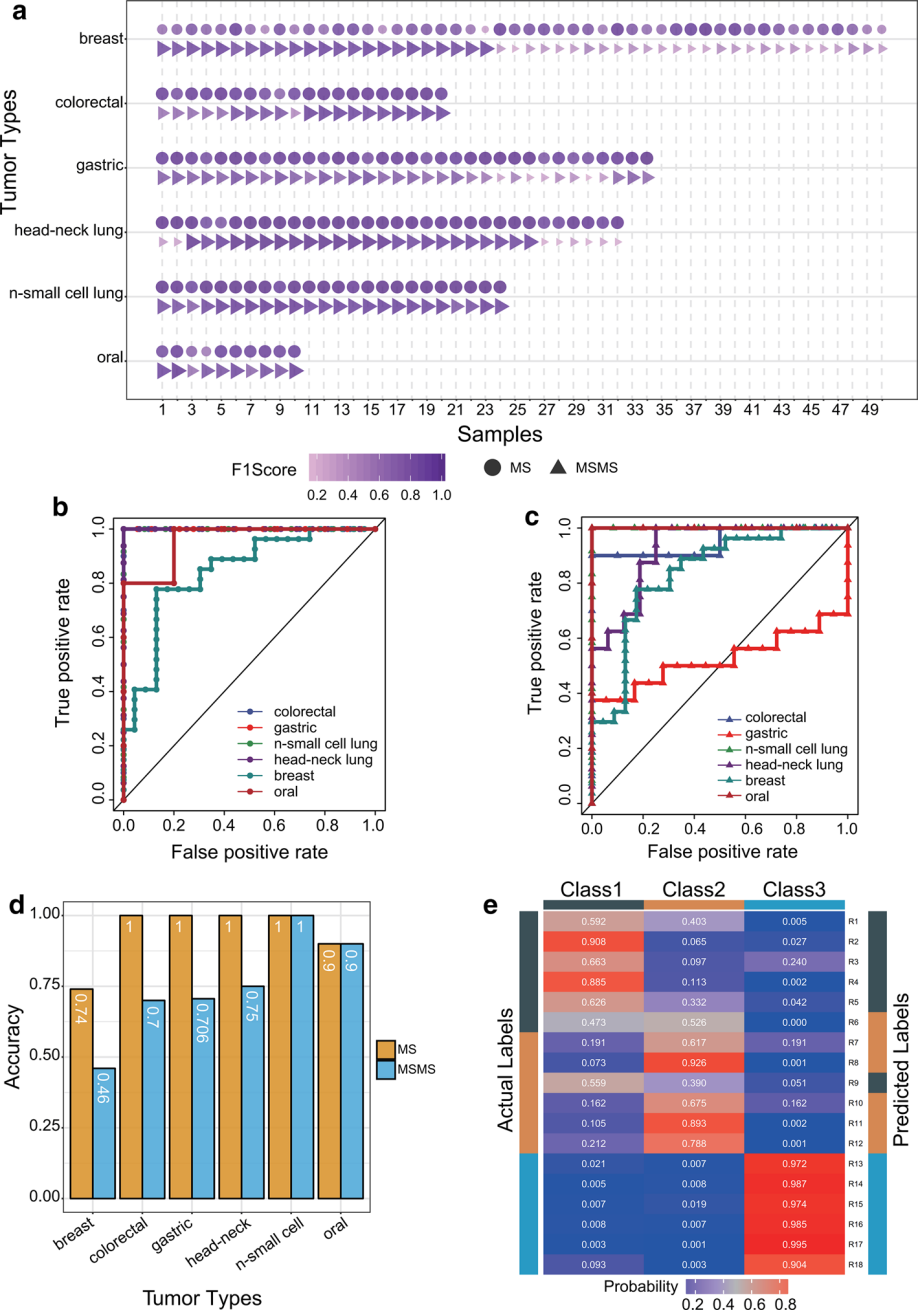
**a** F1-score distribution of MS1 mass spectra (marked with dots) and MS2 mass spectra (marked with triangles) across every sample. **b** ROC curves for each tumor type calculated using MS1 data and **c** MS2 data. **d** Accuracy of the data prediction of every tu-mor type. **e** Heatmap of the multiclassification prediction probability of every sample (Class 1: Normal samples; Classes 2 and 3: two different treatment conditions of nonsmall cell lung cancer samples)

After selection of a suitable window size and determination of pattern difference, we started analyzing the data of all six tumor types independently. The performance of MSpectraAI is mainly demonstrated by the following three factors: (1) F1 score, (2) ROC curve, and (3) multiclassification. (1) F1 score is used as a measure of every sample accuracy. The bubble plot in Fig. 5a shows the F1-score distribution of MS1 mass spectra (marked with dots) and MS2 mass spectra (marked with triangles) in every tumor type. As shown, the colors and sizes of the dots are approximate or superior to those of the triangles, indicating that the prediction of every sample is more accurate when using MS1 data. (2) ROC curve measures the performance of the classification problem at various threshold settings, and was analyzed for each tumor type based on MS1 data (Fig. 5b) and MS2 data (Fig. 5c). It was calculated using the same constructed DNN classification model. All AUC values deduced from MS1 data (average 0.967) were larger than those deduced from MS2 data (average 0.872). Figure 5d shows the prediction accuracy of every tumor type data, confirming that MS1 spectra may contain more information of the tumor. (3) MSpectraAI also supports users to analyze multiclass samples rather than just a two-category problem. The heat map in Fig. 5e displays the prediction probability of every sample calculated using MS1 data in one untreated state (Class 1) and two drug-treatment states of nonsmall cell lung cancer (Classes 2 and 3) [28]. The overall accuracy is 0.89 across all samples, whereas the prediction probabilities of two mispredicted samples are relatively close to those of actual labels. For example, the actual label of sample R6 is Class 1 while the predicted label fell into Class 2 (0.526 versus 0.437 probabilities in Class 1), which implies that the MS data-acquisition method or DNN models may need to be optimized repeatedly for such complicated samples.

Additionally, on the one hand, from the comparison results among MSpectraAI and other machine-learning algorithms (Table 2), the accuracy, as well as relative sensitivity, precision, and F1 score from MSpectraAI based on MS1 data are generally higher than those obtained from published results in which the authors selected the identified proteins as model features to distinguish or predict the normal and cancer samples [25], on the other hand, when compared with the ‘MaxQuant + DNN’ mode, all results (Table 3) demonstrate that MSpectraAI still keeps a reasonably similar or even superior performance in the prediction of complex clinical samples (except the colorectal cancer datasets because of the low quality of protein matrix data).

**Table 2:** Performance comparison among MSpectraAI and other diverse types of machine-learning algorithms on different datasets

	Oral cancer dataset					Head-and-neck dataset	
	Random Forest	Logistic Regression	Linear SVM	RBF SVM	MSpectraAI	Linear SVM	MSpectraAI
Accuracy	0.674	0.594	0.573	0.540	0.90	0.868	1.00
Sensitivity	0.751	0.811	0.809	0.799	1.00	0.85	1.00
Precision	0.753	0.654	0.638	0.615	0.833	0.889	1.00
F1	0.751	0.724	0.713	0.695	0.909	0.895	1.00

**Table 3:** Performance comparison between MSpectraAI and the classic approach utilizing MaxQuant + DNN mode on diverse datasets

	Method	PXD007232 [25]	PXD008012 [26]	PXD007705 [27]	PXD005698 [28]	PXD002213 [29]	PXD009602 [30]
Accuracy	Classic	0.70	0.52	0.969	0.625	0.794	–
	MSpectraAI	0.90	0.74	1.00	1.00	1.00	1.00
Sensitivity	Classic	0.75	0.551	1.00	0.615	0.765	–
	MSpectraAI	1.00	0.727	1.00	1.00	1.00	1.00
Precision	Classic	0.60	0.593	0.938	0.667	0.813	–
	MSpectraAI	0.833	0.696	1.00	1.00	1.00	1.00
F1	Classic	0.667	0.571	0.968	0.64	0.788	–
	MSpectraAI	0.909	0.711	1.00	1.00	1.00	1.00

“Classic” in the Method means the classic approach- “MaxQuant + DNN” mode; –, means no value

## Discussion

The conventional data-analysis strategy is concerned about the exact protein expression in biological samples, providing detailed information for the biological process and pathway analysis [33, 34]. These decoded proteins are very useful for elucidating the biological mechanism and discovering the biomarkers for sample classification [35]. However, protein identification, functional analysis, and validation are time-limiting steps for downstream application of proteomics. MSpectraAI can make full use of ten to hundred thousands of multidimensional spectral features in each acquired raw file without decoding them into proteins but making an accurate classification. Thus, MSpectraAI shows great potential in precision medicine including disease screening, diagnosis, prognosis, responses to treatment, and health management. Moreover, although all data from ProteomeXchange were acquired from different laboratories with different sample preparation procedures, MSpectraAI exhibits an excellent flexibility when used with a DNN model to analyze data features and makes accurate predictions compared to those published results (Table 2). Additionally, despite the similar DNN model used, the performance from the ‘MaxQuant + DNN’ mode was worse than that from MSpectraAI (Table 3), which may be due to over-fitting as the dimensionality of protein features (predictors) obtained from MaxQuant was much lower than that derived from raw mass spectra data and suggests that users should consider the problem of over-fitting and assess the impact on prediction accuracy when analyzing low-dimensional data (e.g. proteome intensity matrix data) with a DNN model. Besides these, there are still some limitations of this approach, such as when analyzing large-scale samples, the experiment conditions should be same/consistent throughout the whole research process, e.g. LC conditions including column lot, peek tube, gradient time etc. and mass spectrometer parameters including MS1 range, AGC target, maximum ion injection time etc. (Additional file 1: Table S2), which can affect the consistency of data and the accuracy of this tool prediction. Therefore, it is valuable to point out that when new MS raw data are included into the existing DNN model for prediction analysis in the same case, it is suggested that the MS data should be acquired using the same/consistent LC and MS parameters. As a method of spectra profile recognition, a much shorter LC separation time may be enough to complete the task, thus greatly decreasing the expense and time of MS data collection.

Additionally, users analyzing their own data with MSpectraAI can improve several aspects by themselves. First, modifications can be made in the DDA method, i.e., in each DDA duty cycle of a mass spectrometer, MS2 scans are produced based on varying precursor ions [36, 37], resulting in greater indeterminacy and barely satisfactory results. Therefore, the acquisition of MS2 spectra may not be necessary following each full scan in the DDA model for MSpectraAI analysis. Second, a more eligible MS2 spectra can be acquired for the MSpectraAI program. That is, different data-acquisition methods, such as DIA [24], could deserve to be undertaken for a similar analysis workflow. However, the relative analysis could become more complicated and a more complex algorithm or DNN model may need to be sophisticatedly developed for extracting features or prediction of such data. Third, there is not an algorithm for users to select proper window size automatically in this work. From our results (Fig. 3), nevertheless, we can observe some hints that too small or too large window sizes are not good choice for exploring the difference between normal and cancer samples, which can be as a reference when users analyze their own data. Fourth, multi-classification can be performed for similar type samples. Many diseases can be divided into corresponding subtypes based on certain characteristics; this is highly crucial for sequential treatment and prognosis. MSpectraAI allows users to process multicategory data (Fig. 5e) with respect to the built-in DNN models. However, with the increase in the number of categories, correct prediction would be challenging. Data training for each data category from samples with definite phenotypes would shed light on multi-classification.

## Conclusions

In this study, we develop an open-source and comprehensive platform, named MSpectraAI, for large-scale analysis of raw mass-spectrometric data with deep neural networks. This software can automatically extract and decipher mass spectra profiling using our homemade approach (feature swath extraction) and moreover distinguish the pattern differences with a proper window size between normal and tumor samples, even among multi-label samples, with deep learning method. The results show that MSpectraAI can achieve better prediction accuracy (average 0.967) when using the MS1 spectra than that (average 0.872) when using the MS2 spectra and present a better performance compared to the other classical machine learning approaches. Throughout this work, we anticipate that MSpectraAI could be applied expansively to the analysis of metabolomics or NMR data and could assist relative scientists or clinicians to analyze more complicated samples conveniently with its further development.

## Supplementary information

**Additional file 1**.

## Data Availability

The datasets analyzed during the current study are available in ProteomeXchange consortium, https://www.proteomexchange.org, the PXD IDs are summarized in Additional file 1: Table S1. The source code is available at: https://github.com/wangshisheng/MSpectraAI.

## Abbreviations

LC–MS: Liquid chromatography coupled with mass spectrometry
DDA: Data dependent acquisition
DIA: Data independent acquisition
MS1: Full scan mass spectrum
MS2: Fragment ion scan mass spectrum
kNN: K-nearest neighbor
SVM: Support vector machine
RBF: Radial basis function
ANNs: Artificial neural networks
CNNs: Convolutional neural networks
RNNs: Recurrent neural networks
DNNs: Deep neural networks
ROC: Receiver operating characteristic
AUC: Area under the curve

## Availability and requirements

Project name: MSpectraAI. Project home page: https://github.com/wangshisheng/MSpectraAI. Operating system(s): Windows, Linux, Mac OS. Programming language: R. Other requirements: R packages shiny, keras, ggplot2 etc. License: GNU General Public License V3.0. Any restrictions to use by non-academics: License needed.
