# Supplementary material for: MSpectraAI: a powerful platform for deciphering proteome profiling of multi-tumor mass spectrometry data by using deep neural networks
Source: BMC Bioinformatics. 2020 Oct 7;21:439. doi: 10.1186/s12859-020-03783-0 (PMC7539376; doi:10.1186/s12859-020-03783-0)
Supplement: Supplementary file 1 — Additional file 1. [file 12859_2020_3783_MOESM1_ESM.docx]

***Supporting Information for:***

**MSpectraAI: A powerful platform for deciphering proteome profiling of multi-tumor mass spectrometry data by using deep neural networks**

Shisheng Wang^1,^^†^, Hongwen Zhu^2,†^, Hu Zhou^2^, Jingqiu Cheng^1,^* and Hao Yang^1,^*

^1^ West China-Washington Mitochondria and Metabolism Research Center; Key Lab of Transplant Engineering and Immu-nology, MOH, Regenerative Medicine Research Center, West China Hospital, Sichuan University, Chengdu, China

^2^ Shanghai Institute of Materia Medica, Chinese Academy of Sciences, Shanghai, China

Corresponding Author

*Email address: [yanghao@scu.edu.cn](mailto:yanghao@scu.edu.cn); [jqcheng@scu.edu.cn](mailto:jqcheng@scu.edu.cn).

†Authors contributed equally

**Table of Contents**

**I. Supplementary Figures and Tables**

Figure S1. The logistic flowchart of data analysis.

Figure S2. The deep neural network model for the analysis of multi-tumor mass spectrometry data.

Table S1. The detailed information of raw data collected for data analysis with MSpectraAI.

Table S2. The summary of LC-MS/MS techniques used for every study downloaded from ProteomeXchange consortium.

**II. Supplementary Notes**

1. Brief Description
2. Availability
3. Friendly Tips
4. How to install third-party softwares
5. Installing R packages
6. Browser compatibility
7. Data Preparation
8. Running MSpectraAI locally
9. Operation step by step

**III. Supplementary Methods**

The detailed description about how to generate the proteome matrix data by using MaxQuant for each study collected in this work and process deep neural network model for the classifications.

**IV. References**

**I. Supplementary Figures and Tables**


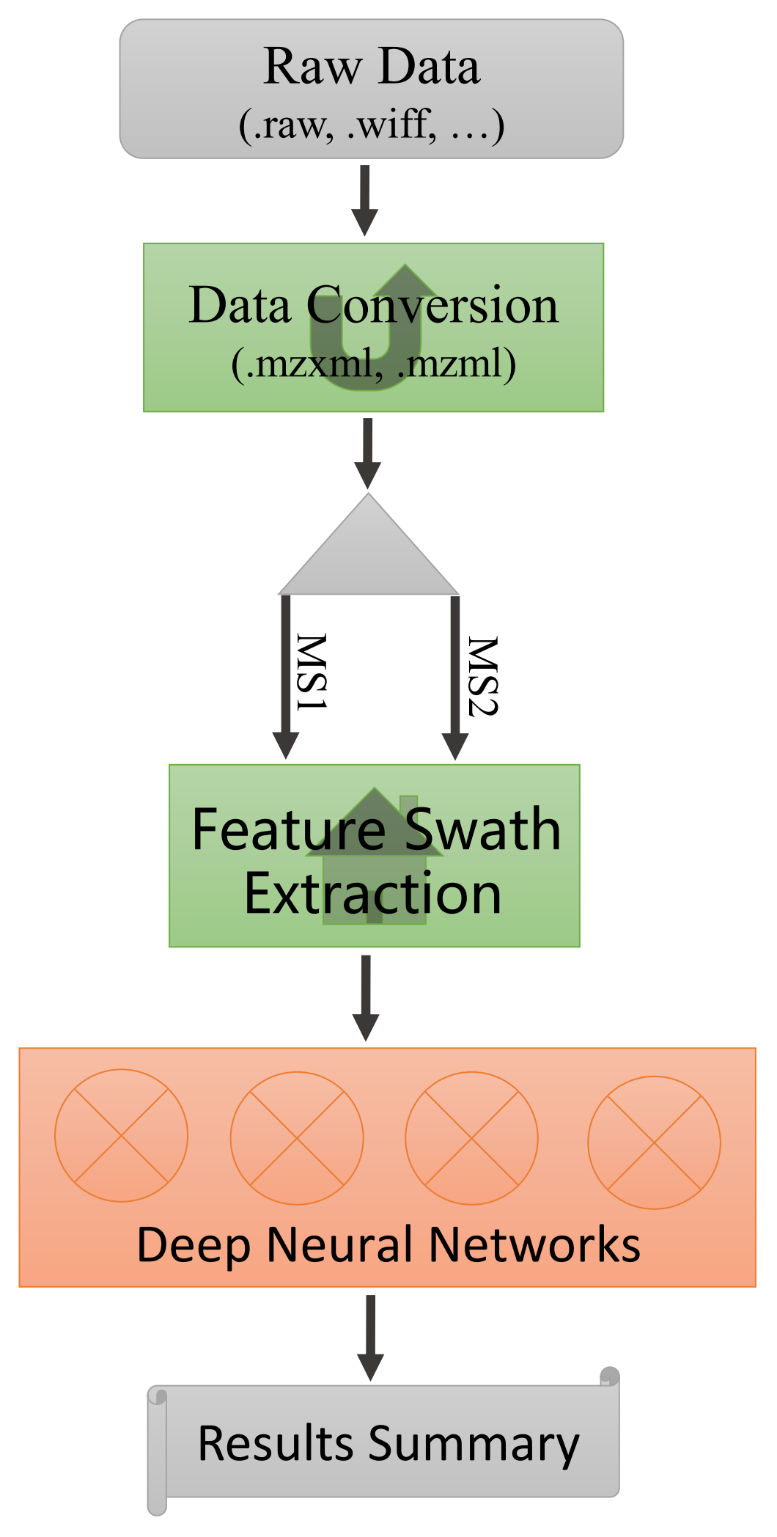


**Figure S1.** The logistic flowchart of data analysis. Raw mass-spectra data are converted into mzXML or mzML format, and then features are extracted from MS1 and MS2 spectra, respectively, for the following DNN model to obtain the results.


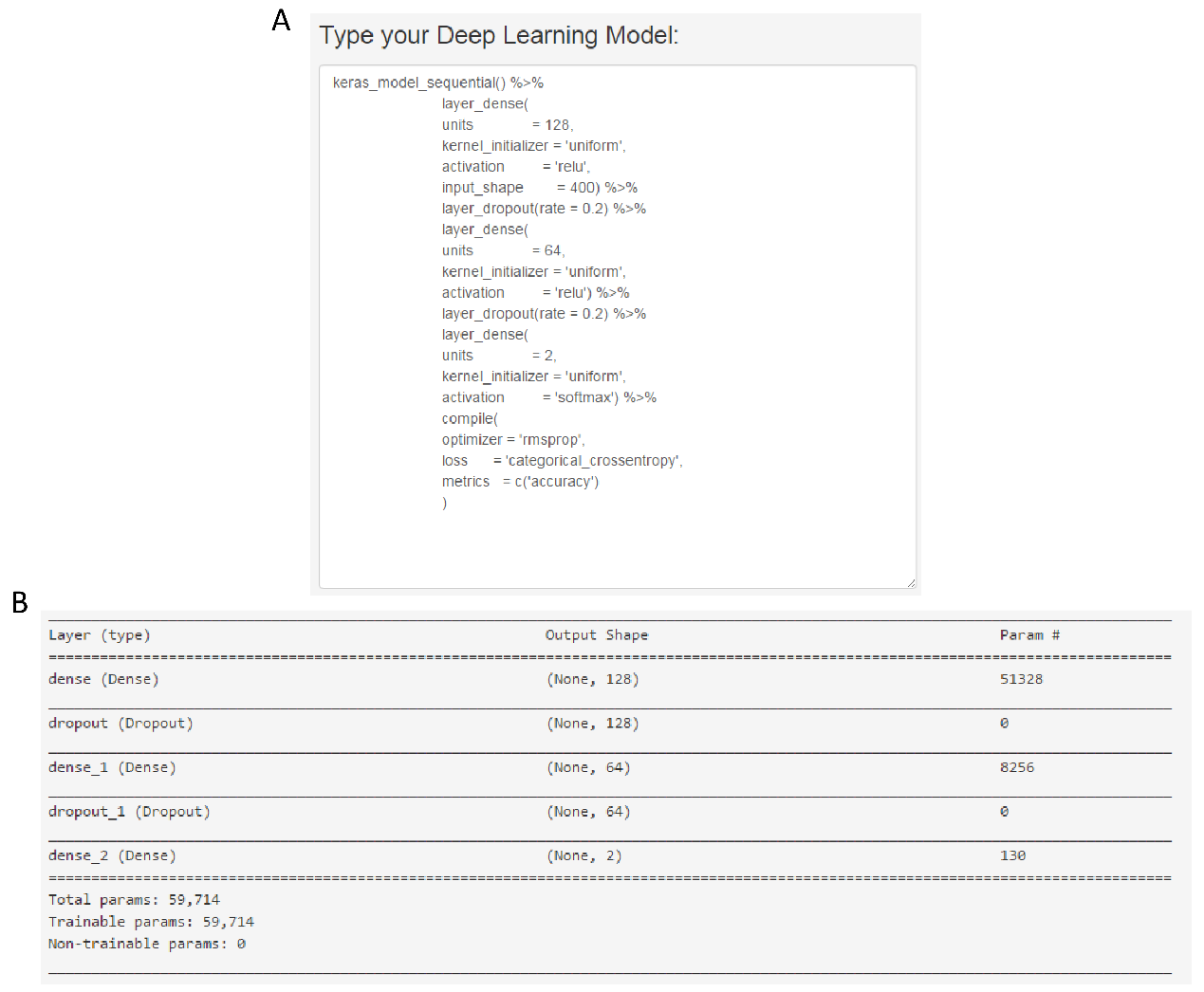


**Figure S2**. The deep neural network model built in MSpectraAI for the analysis of multi-tumor mass spectrometry data. A. The original R codes for binary classification. The code (keras_model_sequential()) in fist row means initializing the DNN model, layer_dense() means adding a densely-connected NN layer to an output, layer_dropout() means applying dropout to the input. Here we totally build a three-layer DNN model. B. The summary of the deep neural network model built in A. From the output, we know there are total 59,714 trainable parameters. The model can also be changed by users in “Deep Learning Model” part in MSpectraAI.

**Table S1.** The detailed information of raw data collected for data analysis with MSpectraAI

| Names | PXD | Filenames |
| --- | --- | --- |
| colorectal cancer | PXD009602 | UPN_11_HLA-class_II_CRC_malignant_Rep231.raw |
| colorectal cancer | PXD009602 | UPN_11_HLA-class_II_CRC_malignant_Rep232.raw |
| colorectal cancer | PXD009602 | UPN_11_HLA-class_II_CRC_malignant_Rep233.raw |
| colorectal cancer | PXD009602 | UPN_11_HLA-class_II_CRC_malignant_Rep234.raw |
| colorectal cancer | PXD009602 | UPN_11_HLA-class_II_CRC_malignant_Rep235.raw |
| colorectal cancer | PXD009602 | UPN_11_HLA-class_II_NMT_benign_Rep231.raw |
| colorectal cancer | PXD009602 | UPN_11_HLA-class_II_NMT_benign_Rep232.raw |
| colorectal cancer | PXD009602 | UPN_11_HLA-class_II_NMT_benign_Rep233.raw |
| colorectal cancer | PXD009602 | UPN_11_HLA-class_II_NMT_benign_Rep234.raw |
| colorectal cancer | PXD009602 | UPN_11_HLA-class_II_NMT_benign_Rep235.raw |
| colorectal cancer | PXD009602 | UPN_13_HLA-class_II_CRC_malignant_Rep231.raw |
| colorectal cancer | PXD009602 | UPN_13_HLA-class_II_CRC_malignant_Rep232.raw |
| colorectal cancer | PXD009602 | UPN_13_HLA-class_II_CRC_malignant_Rep233.raw |
| colorectal cancer | PXD009602 | UPN_13_HLA-class_II_CRC_malignant_Rep234.raw |
| colorectal cancer | PXD009602 | UPN_13_HLA-class_II_CRC_malignant_Rep235.raw |
| colorectal cancer | PXD009602 | UPN_13_HLA-class_II_NMT_benign_Rep231.raw |
| colorectal cancer | PXD009602 | UPN_13_HLA-class_II_NMT_benign_Rep232.raw |
| colorectal cancer | PXD009602 | UPN_13_HLA-class_II_NMT_benign_Rep233.raw |
| colorectal cancer | PXD009602 | UPN_13_HLA-class_II_NMT_benign_Rep234.raw |
| colorectal cancer | PXD009602 | UPN_13_HLA-class_II_NMT_benign_Rep235.raw |
| gastric cancer | PXD002213 | 20121023_N_18_19_1.raw |
| gastric cancer | PXD002213 | 20121023_N_18_19_2.raw |
| gastric cancer | PXD002213 | 20121023_N_18_19_3.raw |
| gastric cancer | PXD002213 | 20121023_N_20_1.raw |
| gastric cancer | PXD002213 | 20121023_N_20_2.raw |
| gastric cancer | PXD002213 | 20121023_N_20_3.raw |
| gastric cancer | PXD002213 | 20121023_N_21_1.raw |
| gastric cancer | PXD002213 | 20121023_N_21_2.raw |
| gastric cancer | PXD002213 | 20121023_N_21_3.raw |
| gastric cancer | PXD002213 | 20121023_N4_22_1.raw |
| gastric cancer | PXD002213 | 20121023_N4_22_2.raw |
| gastric cancer | PXD002213 | 20121023_N4_22_3.raw |
| gastric cancer | PXD002213 | 20121023_N4_24_25_1.raw |
| gastric cancer | PXD002213 | 20121023_N4_24_25_2.raw |
| gastric cancer | PXD002213 | 20121023_N4_24_25_3.raw |
| gastric cancer | PXD002213 | 20121023_N4_28_29_1.raw |
| gastric cancer | PXD002213 | 20121023_N4_28_29_2.raw |
| gastric cancer | PXD002213 | 20121023_N4_28_29_3.raw |
| gastric cancer | PXD002213 | 20121024_C_18_19_1.raw |
| gastric cancer | PXD002213 | 20121024_C_18_19_2.raw |
| gastric cancer | PXD002213 | 20121024_C_18_19_3.raw |
| gastric cancer | PXD002213 | 20121024_C_20_1.raw |
| gastric cancer | PXD002213 | 20121024_C_20_2.raw |
| gastric cancer | PXD002213 | 20121024_C_20_3.raw |
| gastric cancer | PXD002213 | 20121024_C_21_1.raw |
| gastric cancer | PXD002213 | 20121024_C_21_3.raw |
| gastric cancer | PXD002213 | 20121024_C_22_1.raw |
| gastric cancer | PXD002213 | 20121024_C_22_2.raw |
| gastric cancer | PXD002213 | 20121024_C_22_3.raw |
| gastric cancer | PXD002213 | 20121027_C_24_25_2.raw |
| gastric cancer | PXD002213 | 20121027_C_24_25_3.raw |
| gastric cancer | PXD002213 | 20121027_C_28_29_1.raw |
| gastric cancer | PXD002213 | 20121027_C_28_29_2.raw |
| gastric cancer | PXD002213 | 20121027_C_28_29_3.raw |
| non-small cell lung | PXD005698 | C1_A_H358%20CAP-40-2156.raw |
| non-small cell lung | PXD005698 | C1_B_H358%20CAP-40-2158.raw |
| non-small cell lung | PXD005698 | C2_A_H358%20CAP-40-2161.raw |
| non-small cell lung | PXD005698 | C2_B_H358%20CAP-40-2164.raw |
| non-small cell lung | PXD005698 | C3_A_H358%20CAP-40-2173.raw |
| non-small cell lung | PXD005698 | C3_B_H358%20CAP-40-2177.raw |
| non-small cell lung | PXD005698 | D1_A_H358%20CAP-40-2180.raw |
| non-small cell lung | PXD005698 | D1_B_H358%20CAP-40-2183.raw |
| non-small cell lung | PXD005698 | D2_A_H358%20CAP-40-2186.raw |
| non-small cell lung | PXD005698 | D2_B_H358%20CAP-40-2189.raw |
| non-small cell lung | PXD005698 | D3_A_H358%20CAP-40-2192.raw |
| non-small cell lung | PXD005698 | D3_B_H358%20CAP-40-2195.raw |
| non-small cell lung | PXD005698 | Narla_794_A549_C1_1_073013.raw |
| non-small cell lung | PXD005698 | Narla_794_A549_C1_2_073013.raw |
| non-small cell lung | PXD005698 | Narla_794_A549_C2_1_073013.raw |
| non-small cell lung | PXD005698 | Narla_794_A549_C2_2_073013.raw |
| non-small cell lung | PXD005698 | Narla_794_A549_C3_1_073013.raw |
| non-small cell lung | PXD005698 | Narla_794_A549_C3_2_073013.raw |
| non-small cell lung | PXD005698 | Narla_794_A549_D1_1_073013.raw |
| non-small cell lung | PXD005698 | Narla_794_A549_D1_2_073013.raw |
| non-small cell lung | PXD005698 | Narla_794_A549_D2_1_073013.raw |
| non-small cell lung | PXD005698 | Narla_794_A549_D2_2_073013.raw |
| non-small cell lung | PXD005698 | Narla_794_A549_D3_1_073013.raw |
| non-small cell lung | PXD005698 | Narla_794_A549_D3_2_073013.raw |
| head and neck lung | PXD007705 | D_Yepes_010915_120915_FASP_HP10.raw |
| head and neck lung | PXD007705 | D_Yepes_010915_120915_FASP_HP20.raw |
| head and neck lung | PXD007705 | D_Yepes_010915_120915_FASP_HP6.raw |
| head and neck lung | PXD007705 | D_Yepes_061115_271115_HP1.raw |
| head and neck lung | PXD007705 | D_Yepes_061115_271115_R1_HP1.raw |
| head and neck lung | PXD007705 | D_Yepes_240815_240815_R1_HP1.raw |
| head and neck lung | PXD007705 | D_Yepes_240815_240815_R1_HP2.raw |
| head and neck lung | PXD007705 | D_Yepes_240815_240815_R1_HP3.raw |
| head and neck lung | PXD007705 | D_Yepes_240815_240815_R1_HP4.raw |
| head and neck lung | PXD007705 | D_Yepes_240815_260815_R2_HP1.raw |
| head and neck lung | PXD007705 | D_Yepes_240815_260815_R2_HP2.raw |
| head and neck lung | PXD007705 | D_Yepes_240815_260815_R2_HP3.raw |
| head and neck lung | PXD007705 | D_Yepes_240815_260815_R2_HP4.raw |
| head and neck lung | PXD007705 | D_Yepes_260815_270815_HP7.raw |
| head and neck lung | PXD007705 | D_Yepes_260815_280815_R2_HP6.raw |
| head and neck lung | PXD007705 | D_Yepes_260815_280815_R2_HP7.raw |
| head and neck lung | PXD007705 | SQCLC_37_1.raw |
| head and neck lung | PXD007705 | SQCLC_37_2.raw |
| head and neck lung | PXD007705 | SQCLC_38_1.raw |
| head and neck lung | PXD007705 | SQCLC_38_2.raw |
| head and neck lung | PXD007705 | SQCLC_39_1.raw |
| head and neck lung | PXD007705 | SQCLC_39_2.raw |
| head and neck lung | PXD007705 | SQCLC_40_1.raw |
| head and neck lung | PXD007705 | SQCLC_40_2.raw |
| head and neck lung | PXD007705 | SQCLC_41_1.raw |
| head and neck lung | PXD007705 | SQCLC_41_2.raw |
| head and neck lung | PXD007705 | SQCLC_42_1.raw |
| head and neck lung | PXD007705 | SQCLC_42_2.raw |
| head and neck lung | PXD007705 | SQCLC_43_1.raw |
| head and neck lung | PXD007705 | SQCLC_43_2.raw |
| head and neck lung | PXD007705 | SQCLC_44_1.raw |
| head and neck lung | PXD007705 | SQCLC_44_2.raw |
| breast cancer | PXD008012 | 20140108_OR8_RP_HP_B15A.raw |
| breast cancer | PXD008012 | 20140108_OR8_RP_HP_B16A.raw |
| breast cancer | PXD008012 | 20140108_OR8_RP_HP_B3A.raw |
| breast cancer | PXD008012 | 20140108_OR8_RP_HP_B3B.raw |
| breast cancer | PXD008012 | 20140108_OR8_RP_HP_B43A.raw |
| breast cancer | PXD008012 | 20140108_OR8_RP_HP_B4A.raw |
| breast cancer | PXD008012 | 20140108_OR8_RP_HP_B4B.raw |
| breast cancer | PXD008012 | 20140108_OR8_RP_HP_B5A.raw |
| breast cancer | PXD008012 | 20140108_OR8_RP_HP_B5B.raw |
| breast cancer | PXD008012 | 20140108_OR8_RP_HP_B6A.raw |
| breast cancer | PXD008012 | 20140108_OR8_RP_HP_B6B.raw |
| breast cancer | PXD008012 | 20140108_OR8_RP_HP_B9A.raw |
| breast cancer | PXD008012 | 20140108_OR8_RP_HP_norep_B10A.raw |
| breast cancer | PXD008012 | 20140108_OR8_RP_HP_norep_B12A.raw |
| breast cancer | PXD008012 | 20140108_OR8_RP_HP_norep_B13A.raw |
| breast cancer | PXD008012 | 20140108_OR8_RP_HP_norep_B14A.raw |
| breast cancer | PXD008012 | 20140108_OR8_RP_HP_norep_B17A.raw |
| breast cancer | PXD008012 | 20140108_OR8_RP_HP_norep_B18A.raw |
| breast cancer | PXD008012 | 20140108_OR8_RP_HP_norep_B19A.raw |
| breast cancer | PXD008012 | 20140108_OR8_RP_HP_norep_B1B.raw |
| breast cancer | PXD008012 | 20140108_OR8_RP_HP_norep_B20A.raw |
| breast cancer | PXD008012 | 20140108_OR8_RP_HP_norep_B21A.raw |
| breast cancer | PXD008012 | 20140108_OR8_RP_HP_norep_B2A.raw |
| breast cancer | PXD008012 | 20140108_OR8_RP_HP_norep_B2B.raw |
| breast cancer | PXD008012 | 20140108_OR8_RP_HP_norep_B35A.raw |
| breast cancer | PXD008012 | 20140108_OR8_RP_HP_norep_B36A.raw |
| breast cancer | PXD008012 | 20140108_OR8_RP_HP_norep_B37A.raw |
| breast cancer | PXD008012 | 20140108_OR8_RP_HP_norep_B38A.raw |
| breast cancer | PXD008012 | 20140108_OR8_RP_HP_norep_B39A.raw |
| breast cancer | PXD008012 | 20140108_OR8_RP_HP_norep_B42A.raw |
| breast cancer | PXD008012 | 20140113_OR8_RP_HP_B15A_rr.raw |
| breast cancer | PXD008012 | 20140113_OR8_RP_HP_B15B.raw |
| breast cancer | PXD008012 | 20140113_OR8_RP_HP_B15B_rr.raw |
| breast cancer | PXD008012 | 20140113_OR8_RP_HP_B16A_rr.raw |
| breast cancer | PXD008012 | 20140113_OR8_RP_HP_B16B.raw |
| breast cancer | PXD008012 | 20140113_OR8_RP_HP_B44A.raw |
| breast cancer | PXD008012 | 20140113_OR8_RP_HP_B44A_rr.raw |
| breast cancer | PXD008012 | 20140113_OR8_RP_HP_B45A.raw |
| breast cancer | PXD008012 | 20140113_OR8_RP_HP_B45A_rr.raw |
| breast cancer | PXD008012 | 20140113_OR8_RP_HP_B47A.raw |
| breast cancer | PXD008012 | 20140113_OR8_RP_HP_B47A_rr.raw |
| breast cancer | PXD008012 | 20140113_OR8_RP_HP_norep_B10A_rr.raw |
| breast cancer | PXD008012 | 20140113_OR8_RP_HP_norep_B10B.raw |
| breast cancer | PXD008012 | 20140113_OR8_RP_HP_norep_B10B_rr.raw |
| breast cancer | PXD008012 | 20140113_OR8_RP_HP_norep_B12A_rr.raw |
| breast cancer | PXD008012 | 20140113_OR8_RP_HP_norep_B13B.raw |
| breast cancer | PXD008012 | 20140113_OR8_RP_HP_norep_B13B_rr.raw |
| breast cancer | PXD008012 | 20140113_OR8_RP_HP_norep_B14A_rr.raw |
| breast cancer | PXD008012 | 20140113_OR8_RP_HP_norep_B14B.raw |
| breast cancer | PXD008012 | 20140113_OR8_RP_HP_norep_B14B_rr.raw |
| oral cancer | PXD007232 | 03out_CarolCC_IBT0417031_E.raw |
| oral cancer | PXD007232 | 03out_CarolCC_IBT0417031_F.raw |
| oral cancer | PXD007232 | 04out_CarolCC_R8B2741101_E.raw |
| oral cancer | PXD007232 | 04out_CarolCC_R8B2741101_F.raw |
| oral cancer | PXD007232 | 05set_CarolCC_B221946_E.raw |
| oral cancer | PXD007232 | 05set_CarolCC_B221946_F.raw |
| oral cancer | PXD007232 | 07nov_CarolCC_052270R_E.raw |
| oral cancer | PXD007232 | 07nov_CarolCC_052270R_F.raw |
| oral cancer | PXD007232 | 07nov_CarolCC_B303868_E.raw |
| oral cancer | PXD007232 | 07nov_CarolCC_B303868_F.raw |

Table S2. The summary of LC-MS/MS techniques used for every study downloaded from ProteomeXchange consortium.

* CID, collision-induced dissociation; HCD, higher-energy collisional dissociation; - means there is no information found in the published paper.

**II. Supplementary Notes**

1. **Brief Description**

In this study, we presented a free and powerful platform, named MSpectraAI (Mass Spectra Artificial Intelligence), as an easy-to-use stand-alone software for mining and classifying raw LC-MS2-based proteomics or metabolomics data of different samples using deep learning models. Users can also built your own deep neural network model in this software. To date, this platform contains:

1) Feature swath extraction, all collected mass spectra are acquired consistently with sequential windows;

2) Samples classification, different group samples can be tested and predicted using artificial neural networks model;

3) Visualization, the fingerprint of mass spectra and model prediction results are shown as vector graphs or table data.

**2. Availability**

MSpectraAI is an open source web platform, which initiative available in the GitHub repository: <https://github.com/wangshisheng/MSpectraAI>. An example is shown here: <https://www.omicsolution.org/wukong/MSpectraAI/>, to which users can also import their data.

1. **Friendly Tips**

- Run this tool locally. As we know, the raw data from mass spectrometer are usually very large. You can analyze your data on our web server, but the analysis speed will be slower.
- Be familiar with the basic usage of R language. This web tool is developed with R, therefore, if you know some basic knowledge about R, it will help you understand this tool better. However, you need not worry if you know nothing about R, and you can learn to use our tool expertly as well after reading our manual.

1. **How to install third-party softwares**

- Install R. You can download R from here: <https://www.r-project.org/>. We recommend the R version >= 3.5.0.
- Install RStudio (Recommendatory but not necessary). You can download RStudio from here: <https://www.rstudio.com/>. If you decide to use the script editor, we recommend the version >= 1.1.423.
- Install Anaconda (For Windows users). You can download Anaconda from here: <https://www.anaconda.com/download/>.
- Install RawConverter [[7](#_ENREF_7)]. Download from here: <http://fields.scripps.edu/rawconv/>. Optionally, you can also use similar tools, such as MSConvert [[8](#_ENREF_8)], which can be downloaded from here: <http://proteowizard.sourceforge.net/tools.shtml>.

1. **Installing R packages**

We recommend the R version >= 3.5.0. Particularly, if you use unix-like systems, you may need install some dependent packages in advance, for example, on CentOS 7:

sudo yum -y install libxml2-devel igraph-devel libxslt-devel netcdf-devel \

libcurl-devel openssl-devel cairo-devel

pip install virtualenv

##Then open R

#Packages

needpackages<-c("BiocManager","devtools","shiny","shinyjs","shinyBS","ggplot2",

"ggjoy","reshape","openxlsx","gdata","DT","gtools","ggsci","mzR","plyr","tidyr",

"abind","data.table","parallel","ggrastr","ggthemes","viridis",

"glue","ComplexHeatmap","impute","circlize","ROCR","keras")

#Check and install function

CheckInstallFunc <- function(x){

for( i in x ){

# require returns TRUE invisibly if it was able to load package

if(!require(i, character.only = TRUE )){

# If package was not able to be loaded then re-install

install.packages(i, dependencies = TRUE)

if(!require(i, character.only=TRUE)) BiocManager::install(i, dependencies = TRUE)

if(i=="ggrastr"){

devtools::install_github('VPetukhov/ggrastr')

}

}

}

}

#Start to check and install

CheckInstallFunc(needpackages)

#R interface to Keras: https://keras.rstudio.com/

library(keras)

install_keras()

The default installation of Keras is CPU, so you want GPU if your computer supports, you should use this commad: install_keras(tensorflow = "gpu"). And the detailed introduction of GPU installation can be found here: <https://keras.rstudio.com/reference/install_keras.html>.

1. **Browser compatibility**

MSpectraAI can be processed on Windows, Linux, and Mac operating system. We have tested it as this:

| OS | Version | Chrome | Firefox | Safari |
| --- | --- | --- | --- | --- |
| Windows | 7 | 68.0.3440.106 | 63.0.3 | not tested |
| Linux | CentOS 7 | not tested | 52.8.0 | not tested |
| MacOS | HighSierra | 70.0.3538.110 | not tested | 12.0.1 |

1. **Data Preparation**

Users can obtain the mass spectra data in their own laboratory. Otherwise, these raw data can be downloaded from some public database, such as ProteomeXchange Consortium (<http://www.proteomexchange.org/>), where users can search raw data uploaded from other labs across the world. However, users should notice that the ideal raw data are limited and not always found for special analysis. Fortunately, we collected six tumor type data and analysed them with deep neural network model in MSpectraAI. The detailed sample information is listed in supplementary table S1.

In consideration of running speed and time, we reconstruted some small-size raw data from nonsmall cell lung cancer samples. But the whole process of analysis is totally identical in comparison with calculation of large-size data. These small-size raw data were also uploaded to the same github as mentioned above for users to download.

1. **Running MSpectraAI locally**

Once you install R and relative packages well, it would be quite easy to run this tool locally on by two lines of code.

First, download this tool from the github (<https://github.com/wangshisheng/MSpectraAI>), like this:


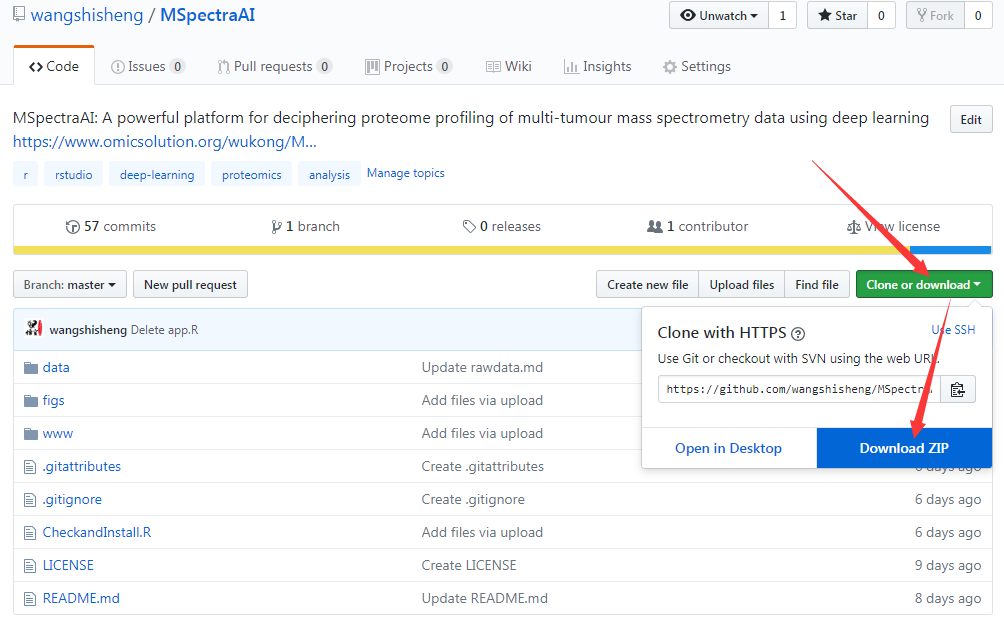


The whole file is about 180MB, so it may take some time.

Second, if you download successfully, unzip this file:


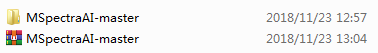


Third, open R-GUI or RStudio. Here, we use RStudio and then find file path, run these codes as below:


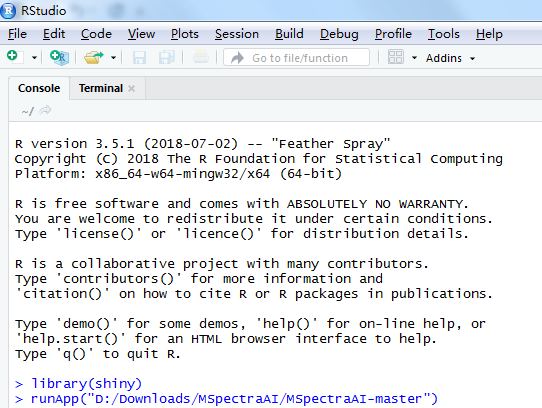


In my computer, the file path is “D:/Downloads/MSpectraAI/MSpectraAI-master”, but yours may be different, so you need change it.

Now, MSpectraAI is activated successfully through listening on a local link. In my computer, it is: <http://127.0.0.1:6201>. Then you can copy this link to a browser, such as Chrome:


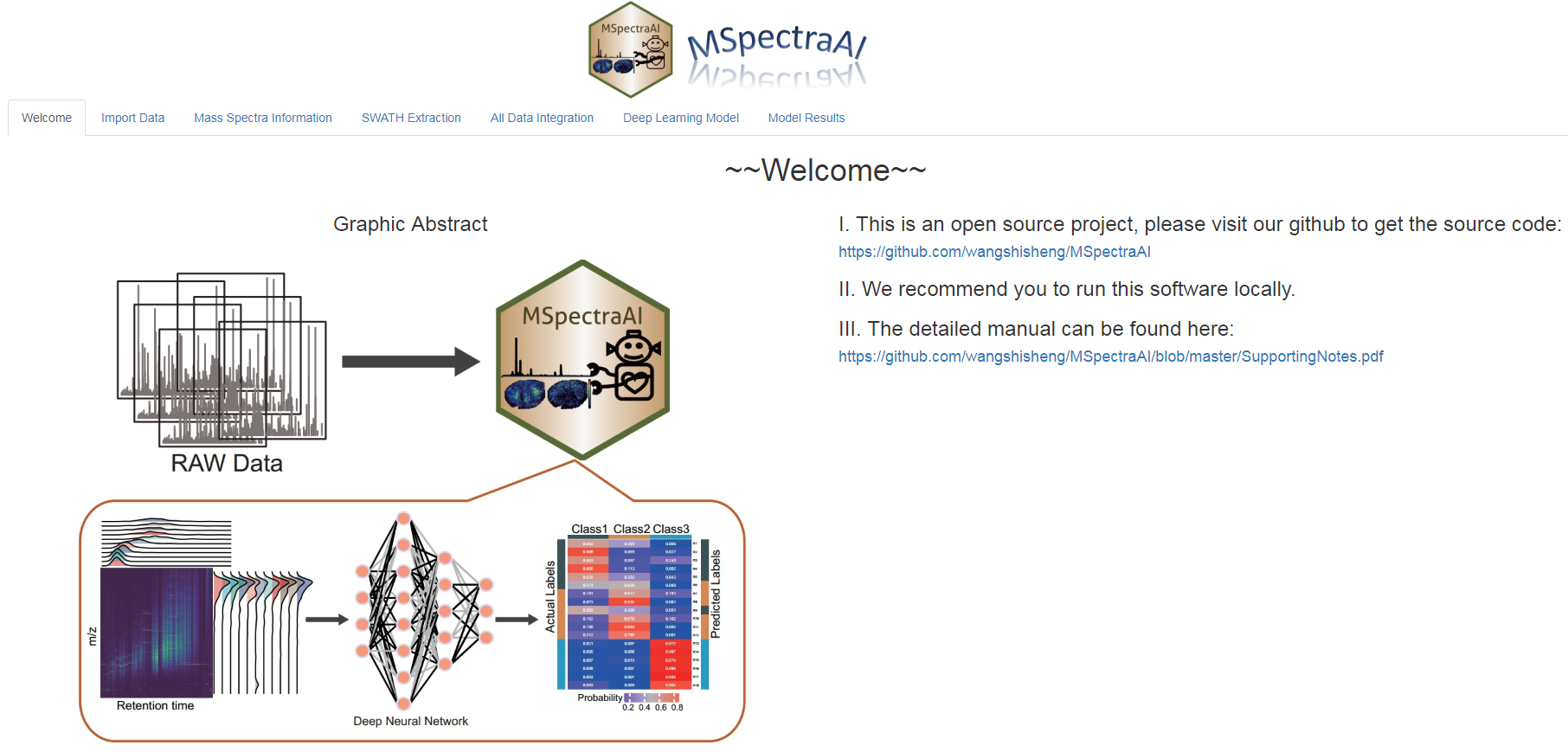


The detailed information about the current R session is shown below:

> sessionInfo()

R version 3.5.1 (2018-07-02)

Platform: x86_64-w64-mingw32/x64 (64-bit)

Running under: Windows 7 x64 (build 7601) Service Pack 1

Matrix products: default

locale:

[1] LC_COLLATE=Chinese (Simplified)_People's Republic of China.936 LC_CTYPE=Chinese (Simplified)_People's Republic of China.936

[3] LC_MONETARY=Chinese (Simplified)_People's Republic of China.936 LC_NUMERIC=C

[5] LC_TIME=Chinese (Simplified)_People's Republic of China.936

attached base packages:

[1] grid parallel stats graphics grDevices utils datasets methods base

other attached packages:

[1] keras_2.1.6 ROCR_1.0-7 gplots_3.0.1 circlize_0.4.4 ComplexHeatmap_1.18.1

[6] glue_1.3.0 viridis_0.5.1 viridisLite_0.3.0 ggthemes_4.0.0 ggrastr_0.1.5

[11] data.table_1.11.8 abind_1.4-5 tidyr_0.8.2 plyr_1.8.4 impute_1.53.0

[16] mzR_2.13.6 Rcpp_0.12.19 ggsci_2.8 gtools_3.5.0 DT_0.4

[21] gdata_2.18.0 openxlsx_4.0.17 ggjoy_0.4.1 ggridges_0.5.0 ggplot2_3.1.0

[26] shinyBS_0.61 shinyjs_1.0 shiny_1.2.0

loaded via a namespace (and not attached):

[1] ProtGenerics_1.11.0 bitops_1.0-6 RColorBrewer_1.1-2 tools_3.5.0 R6_2.2.2 KernSmooth_2.23-15

[7] lazyeval_0.2.1 BiocGenerics_0.26.0 colorspace_1.3-2 GetoptLong_0.1.7 withr_2.1.2 tidyselect_0.2.5

[13] gridExtra_2.3 compiler_3.5.0 Biobase_2.39.2 Cairo_1.5-9 labeling_0.3 caTools_1.17.1.1

[19] scales_1.0.0 tfruns_1.3 stringr_1.3.1 digest_0.6.18 base64enc_0.1-3 pkgconfig_2.0.1

[25] htmltools_0.3.6 htmlwidgets_1.3 rlang_0.3.0.1 GlobalOptions_0.1.0 rstudioapi_0.7 shape_1.4.4

[31] bindr_0.1.1 jsonlite_1.5 tensorflow_1.8 crosstalk_1.0.0 dplyr_0.7.7 magrittr_1.5

[37] Matrix_1.2-14 munsell_0.5.0 reticulate_1.9 stringi_1.1.7 whisker_0.3-2 yaml_2.1.19

[43] promises_1.0.1 crayon_1.3.4 lattice_0.20-35 zeallot_0.1.0 pillar_1.2.1 rjson_0.2.19

[49] codetools_0.2-15 httpuv_1.4.4.1 gtable_0.2.0 purrr_0.2.4.9000 reshape_0.8.7 assertthat_0.2.0

[55] mime_0.5

1. **Operation step by step**
   1. Graphical user interface of MSpectraAI

There are three main parts in this software:

I. Function names. All principle functions are displayed in the menu.

II. Parameter tuning panel. Users can regulate parameters conveniently here according to their own data.

III. Results panel. After uploading data or adjusting parameter, click “Calculate” button, the results will be shown here immediately.


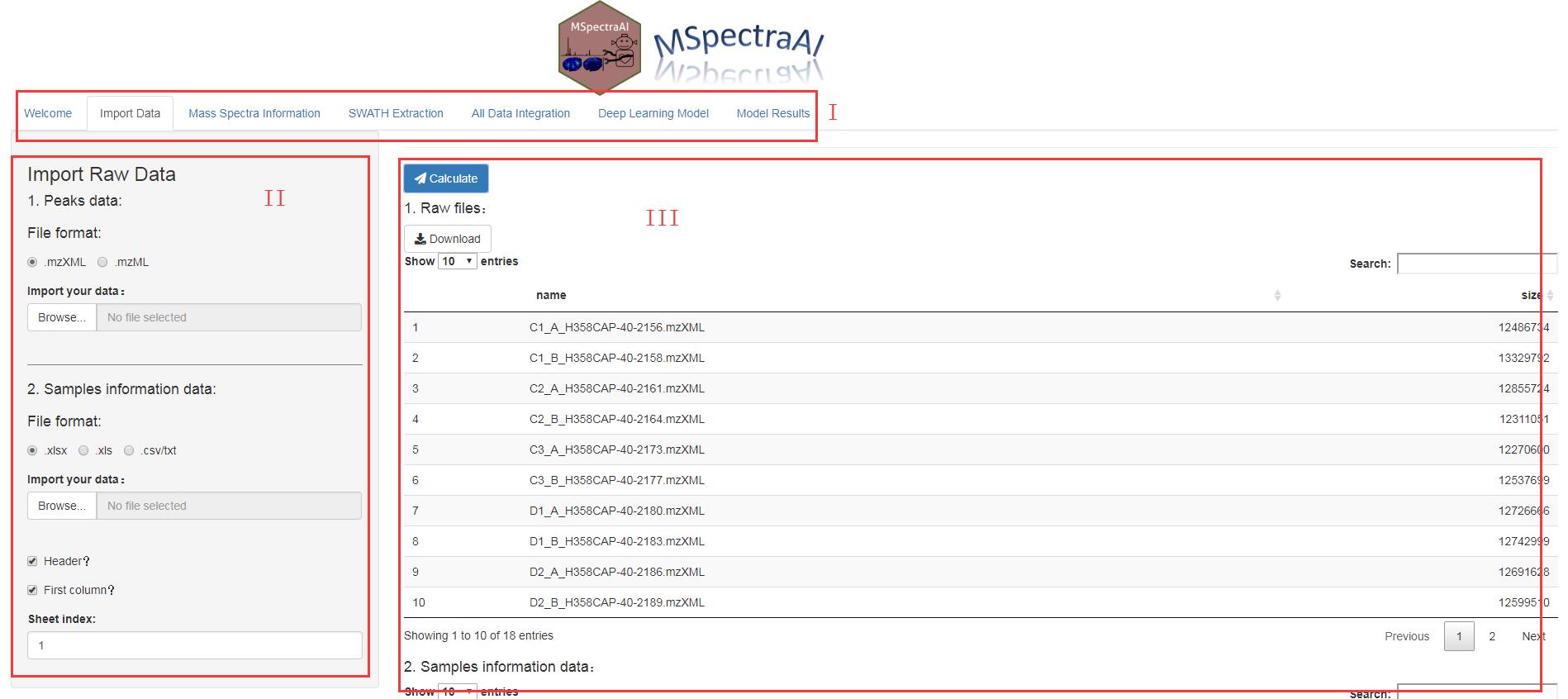


9.2 Importing data

Click “Import Data” name in the menu, then you can upload your data from here. In default, the software will load our example data. Once you upload your own data, the results panel will show the results of your data.


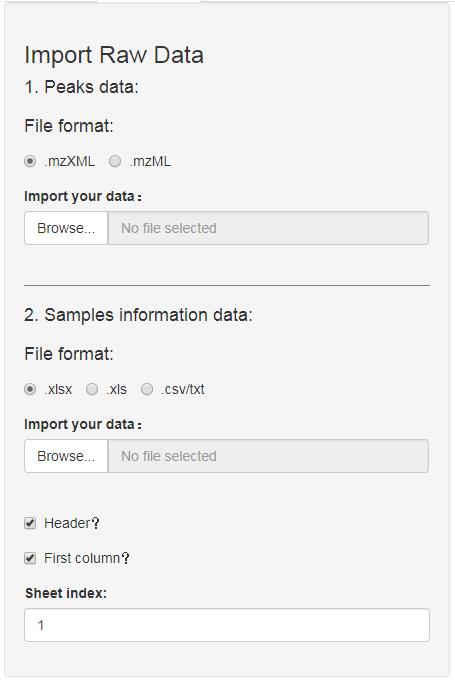


Here you should upload two kinds of data. First, the mzXML or mzML files that converted from raw data using RawConverter or MSConvert software as mentioned above. Second, the sample information data that record the file names and class labels. Once you prepare these data, click “browser”, the results will be shown like this:


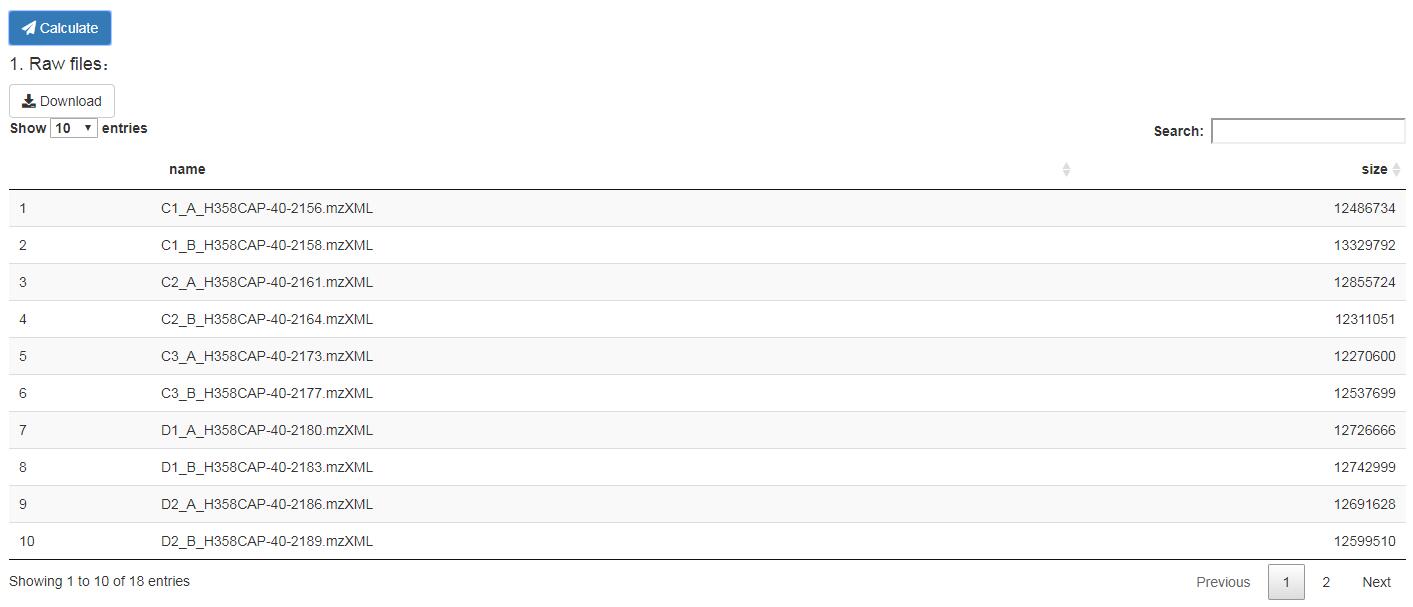


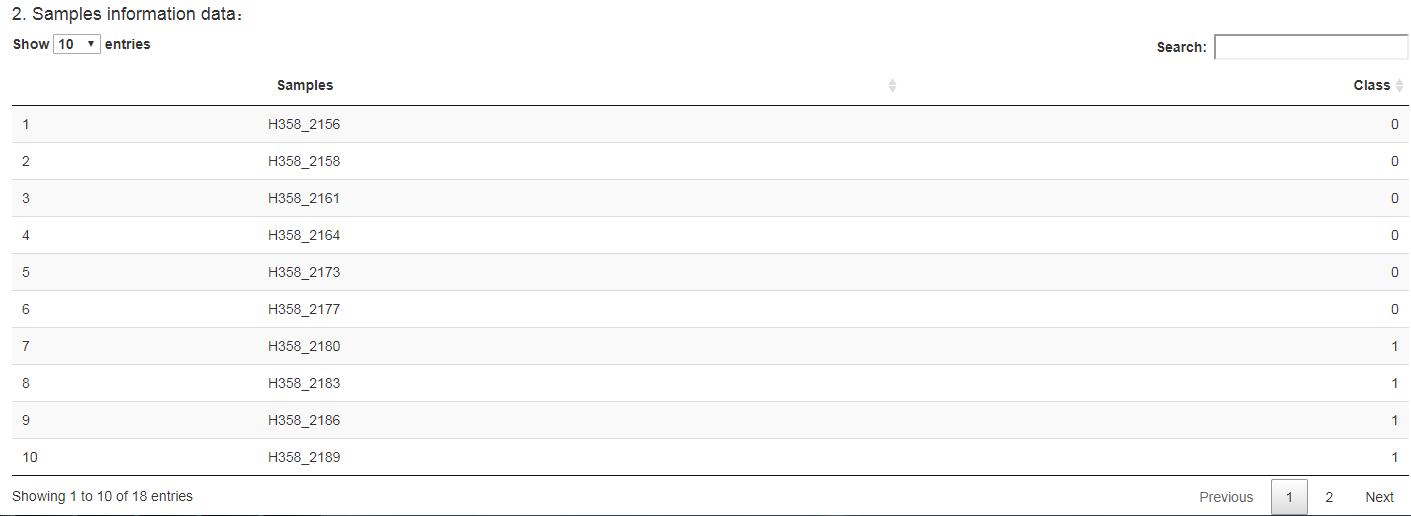


The “name” in “Raw files” result means raw data filenames, “size” means the file size. The “Samples” in “Sample information data” result means sample filenames (also raw data filenames), whose order should be same as raw data filenames. “Class” means category labels, which should be numbers starting from 0.

- 1. Mass Spectra Information

The peaks number in every spectra (shown as histogram), the MS1 spectra number, and the MS2 spectra number (shown in a table) are counted in this part.


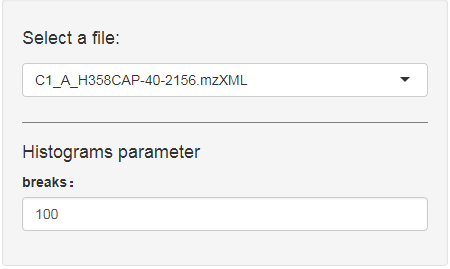


You can select any file and the tool calculates corresponding results immediately. Then click “Download” button, the figures will be saved as pdf files and the tables will be saved as csv files.


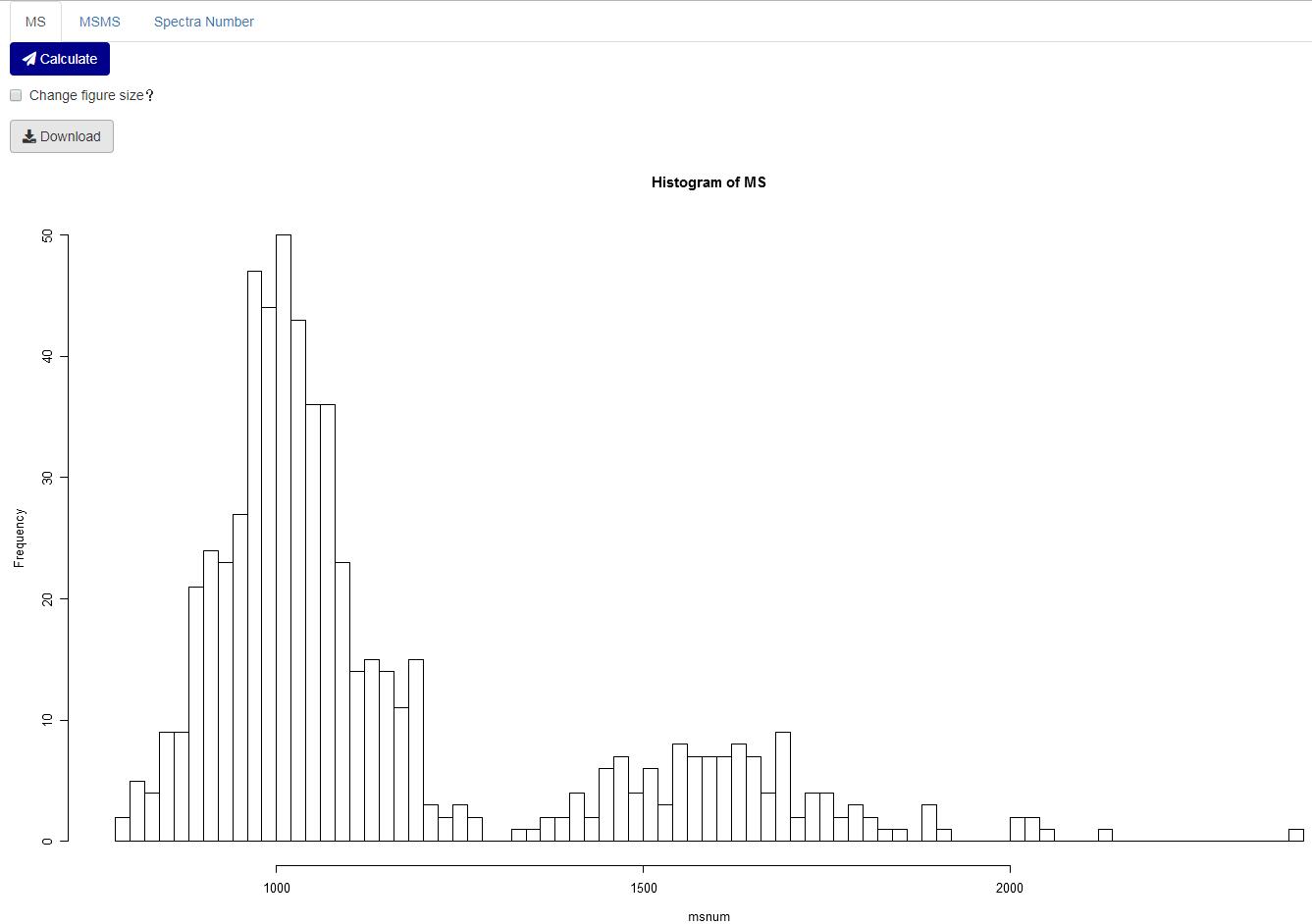


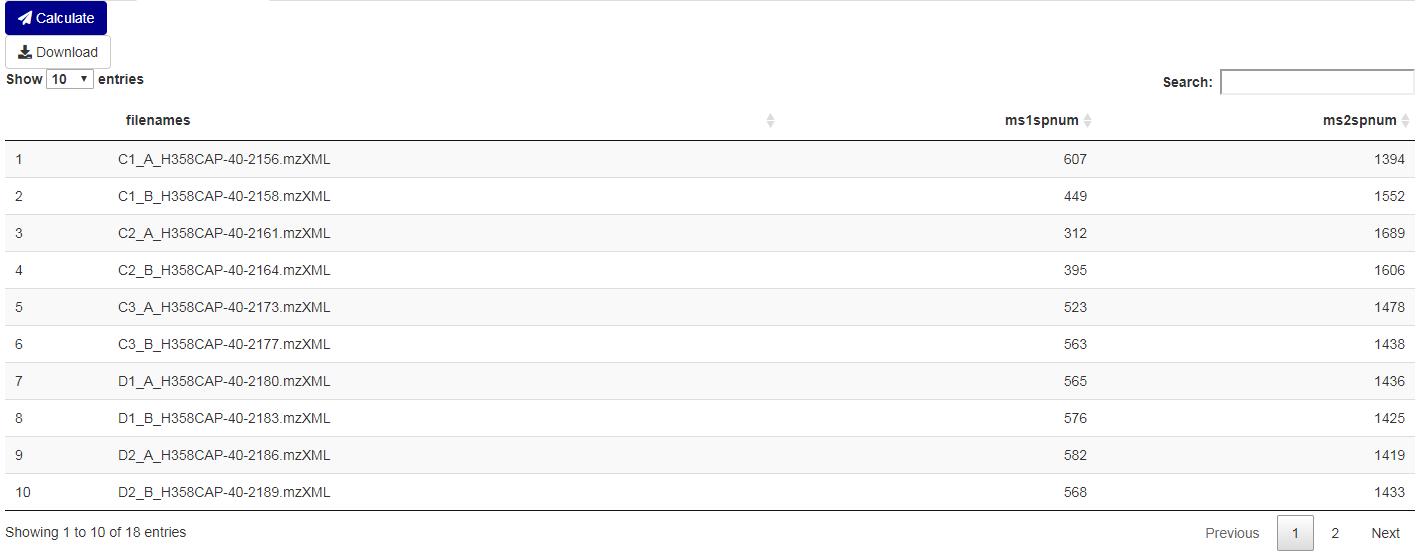


- 1. Swath extraction

Features can be extracted in a certain window size. Parameters can be regulated as below:


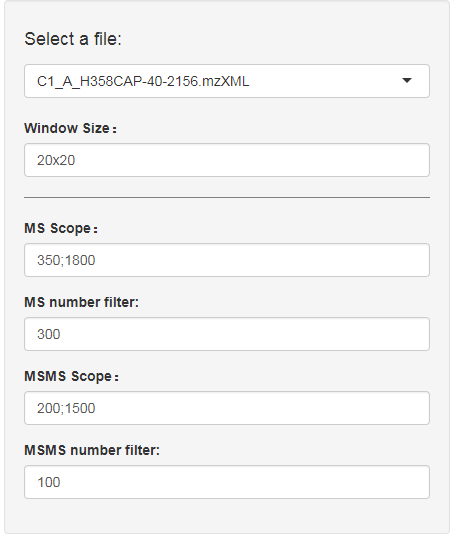


Select a file: users can select a file that they want to analyse.

Window Size: how many windows across the whole *m/z* range. For example, “20x20” means there are total 400 windows and then the whole *m/z* range will be divided into 400 parts.

MS Scope: the *m/z* range of MS scan, which in linked by “;”.

MS number filter: those MS scan whose peaks number are below this threshold will be deleted.

MSMS Scope: the *m/z* range of MS2 scan, which in linked by “;”.

MSMS number filter: those MS2 scan whose peaks number are below this threshold will be deleted.

Then the intensity distribution across *m/z* and retention time dimensions as 2D map and corresponding data matrix table can be calculated here:


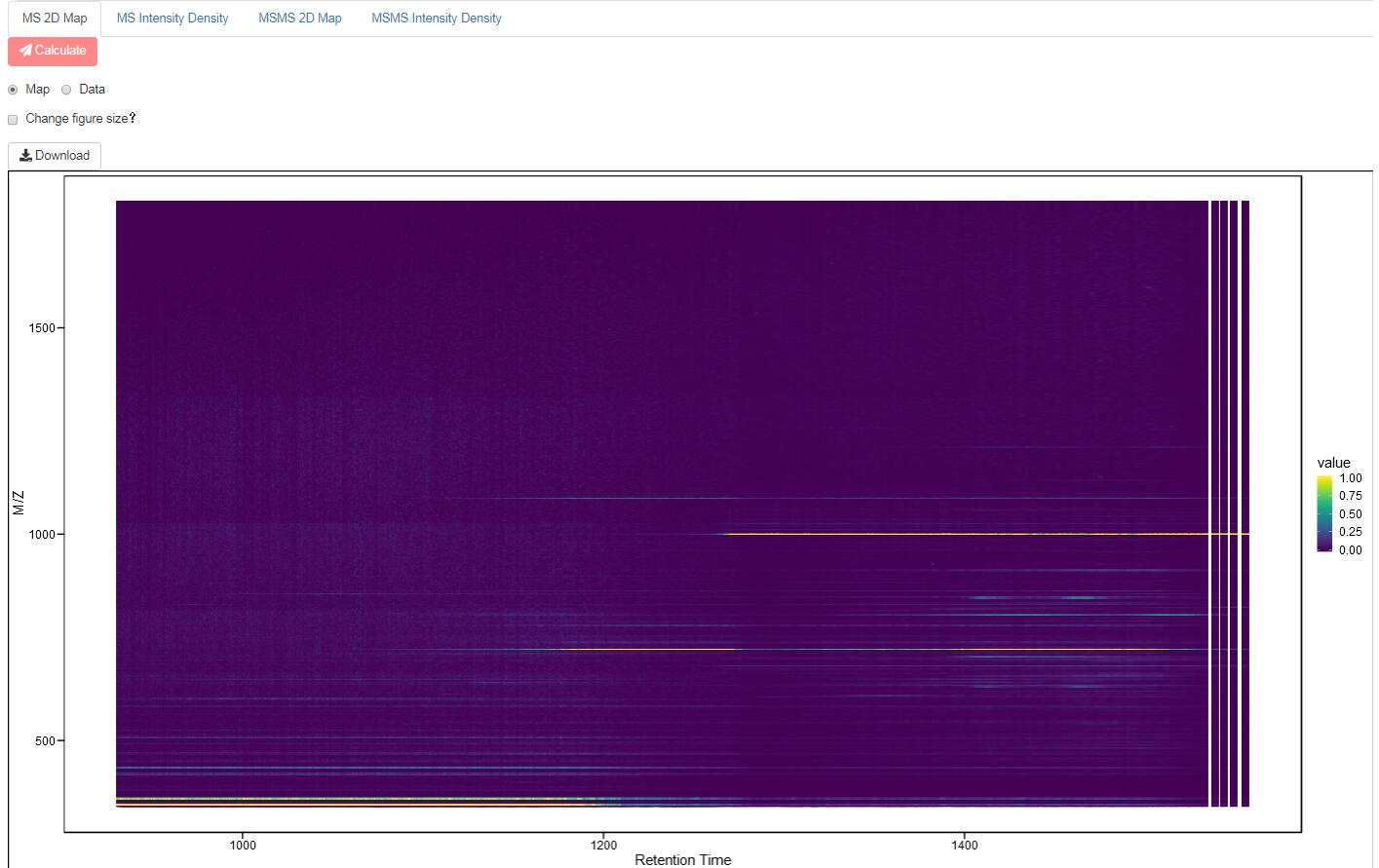


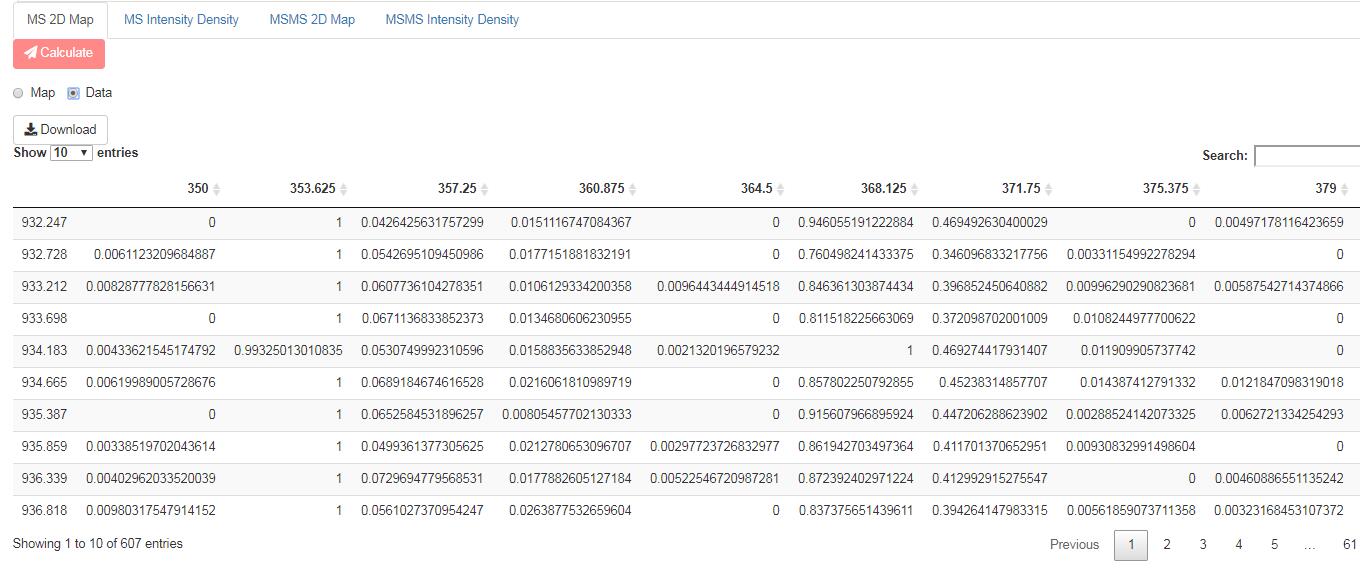


The intensity density can also be displayed here:


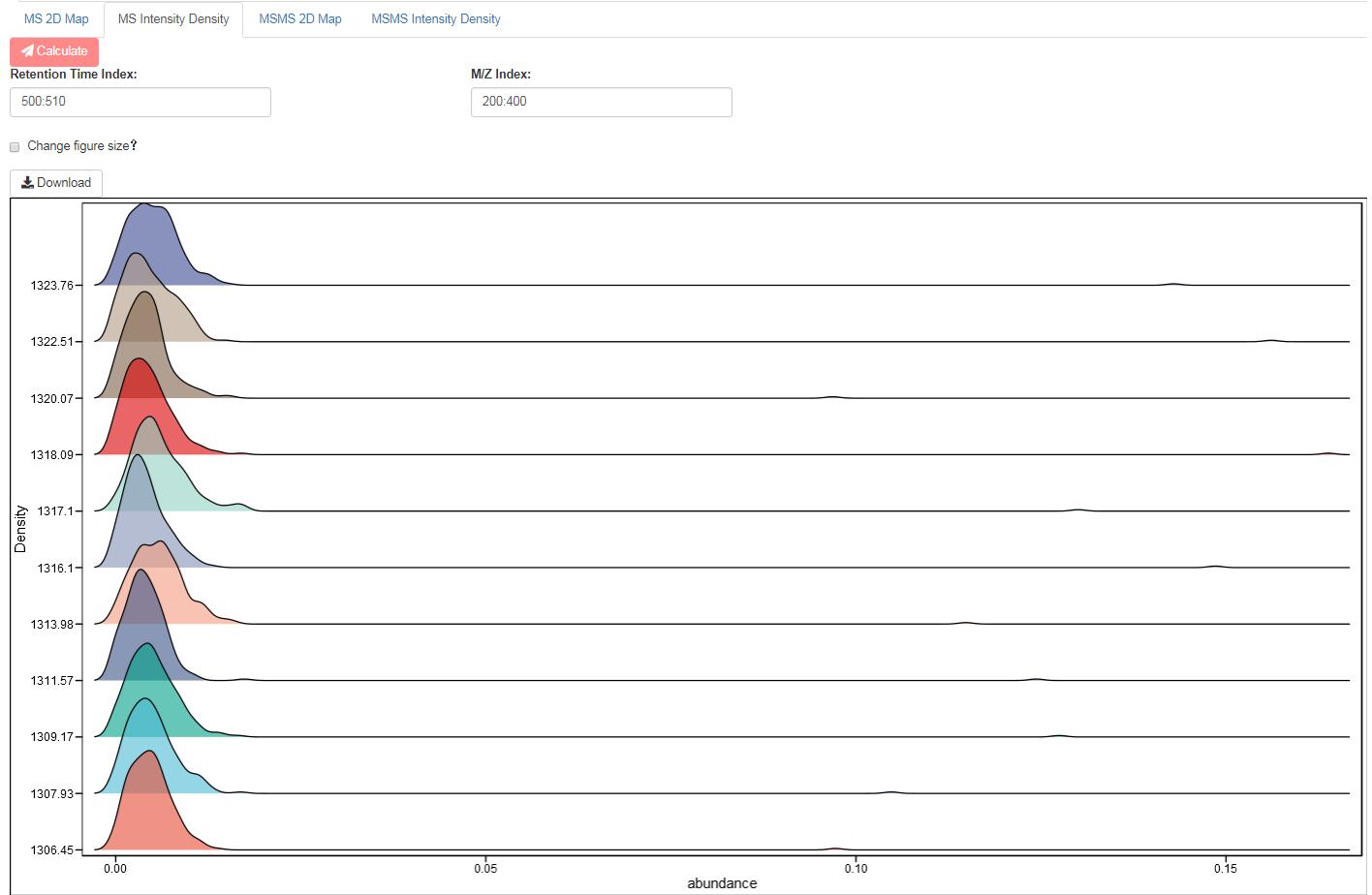


Retention Time Index: which spectra across retention time dimension are extracted to calculate the density.

*M/Z* Index: which spectra across *m/z* dimension are extracted to calculate the density.

All results are similar in MSMS spectra (not shown here).

9.5 All Data Integration

In “9.4 Swath extraction” part, the features are extracted from one file that users select at a time. Here, all files that users upload are extracted and combined together for MS and MS2 spectra data, so the analysis time of this step is a little long. Users should be patient:


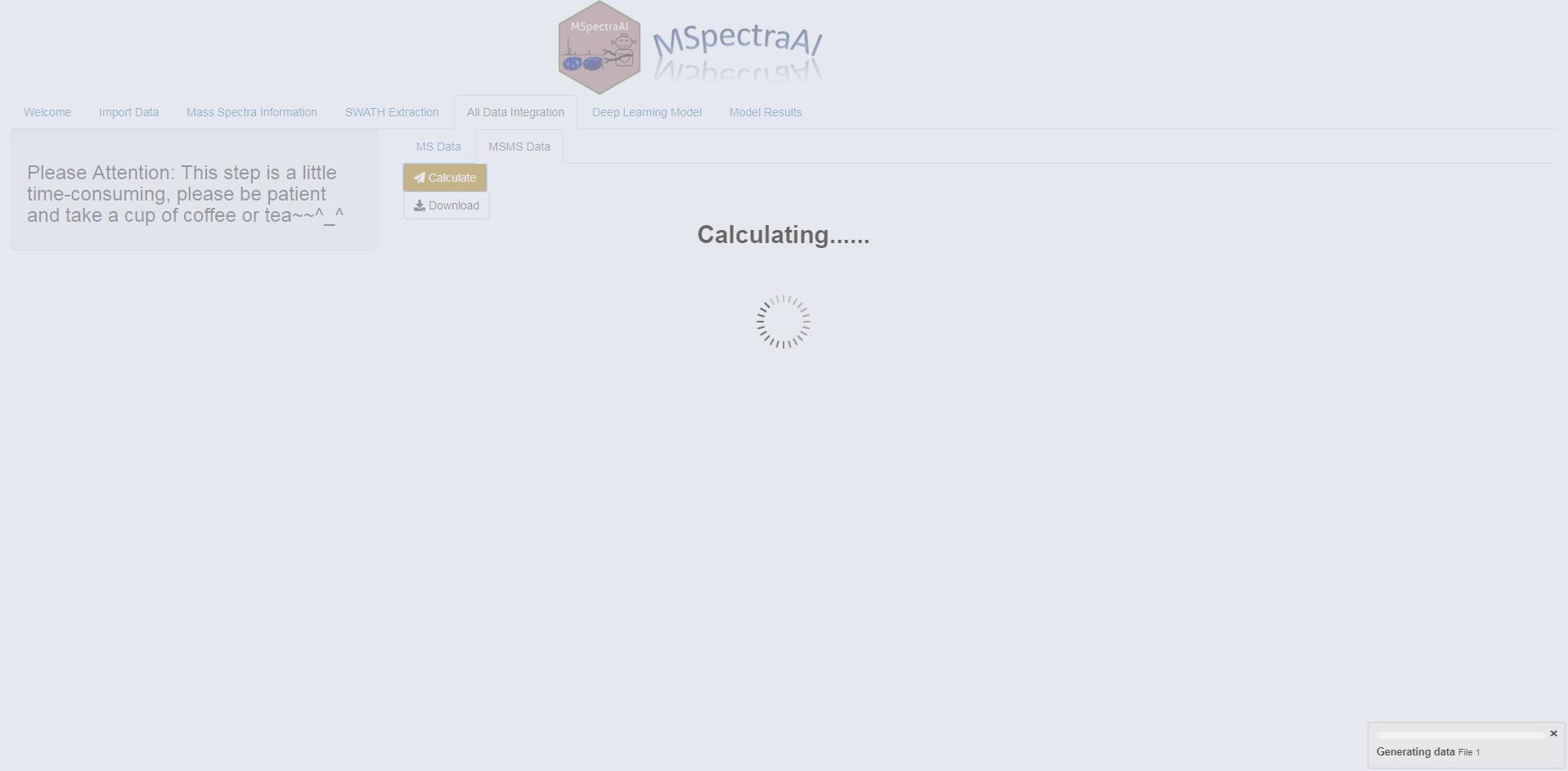


The indicator at the bottom right can tell you which file is processing. And then the whole matrix are shown as below and downloadable by clicking “Download” button:


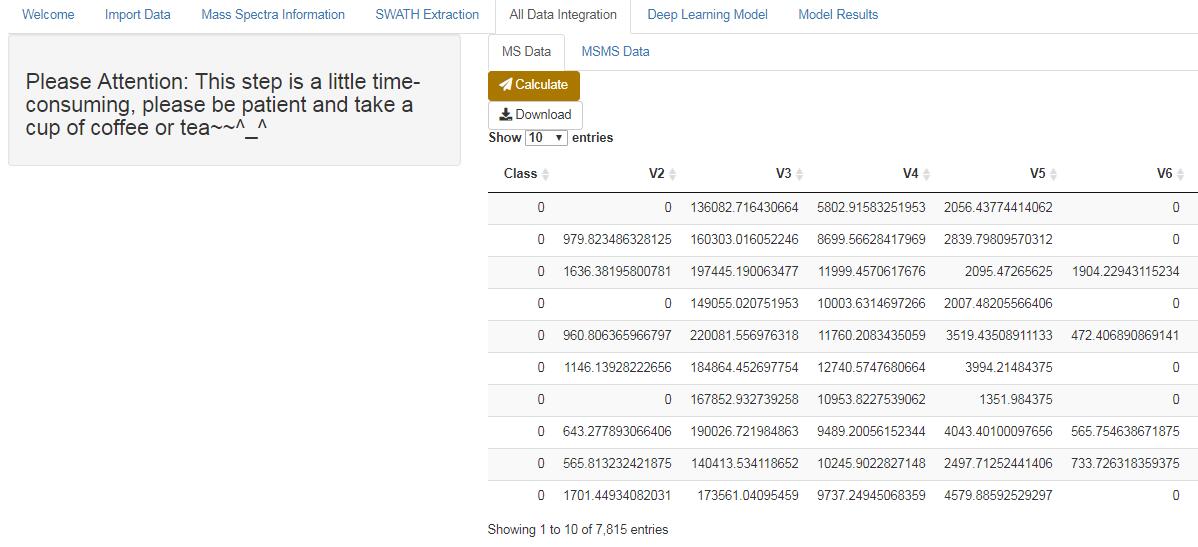


9.6 Deep learning model

In this part, users can obtain the intuition of deep learning model that we build using Keras (<https://github.com/fchollet/keras>) for the example data, the “Model Summary” will give the general information of the deep learning model we input:


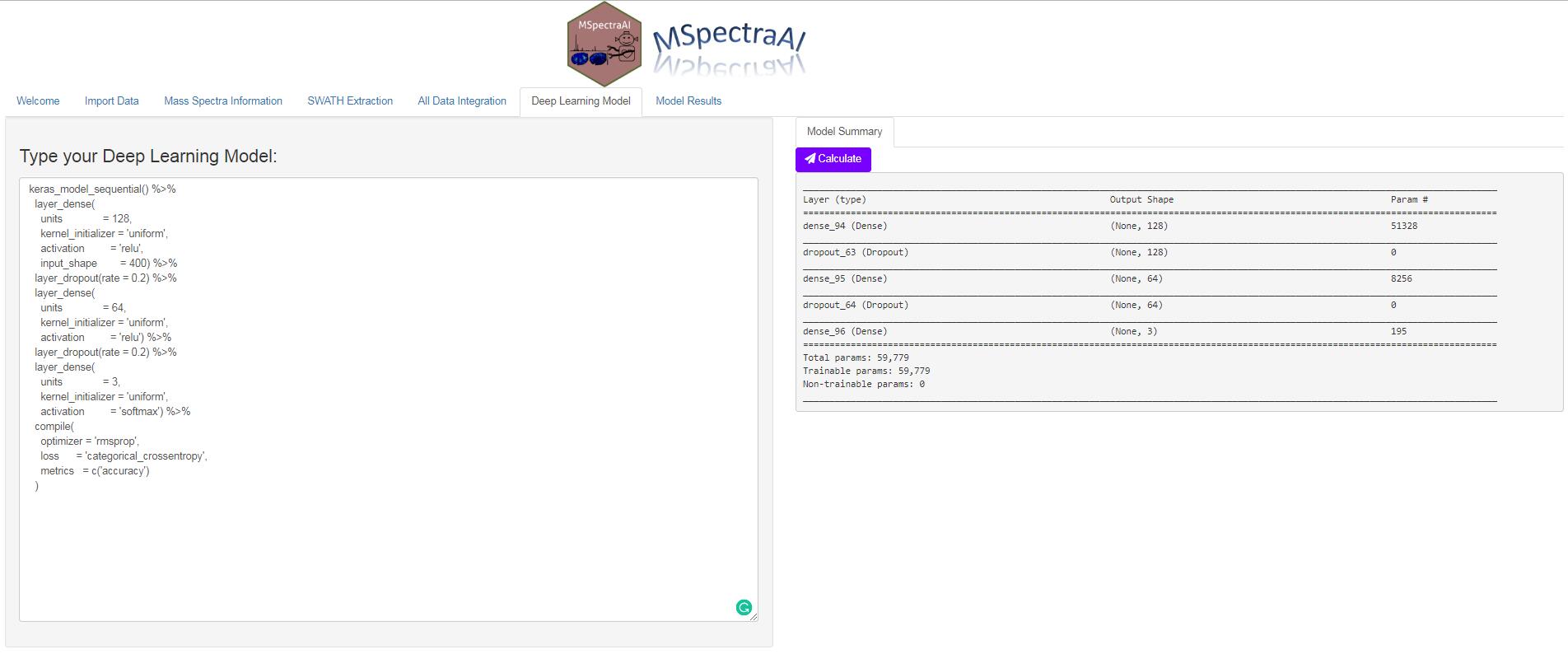


The default code can be changed and shown below:

keras_model_sequential() %>%

layer_dense(

units = 128,

kernel_initializer = 'uniform',

activation = 'relu',

input_shape = 400) %>%

layer_dropout(rate = 0.2) %>%

layer_dense(

units = 64,

kernel_initializer = 'uniform',

activation = 'relu') %>%

layer_dropout(rate = 0.2) %>%

layer_dense(

units = 3,

kernel_initializer = 'uniform',

activation = 'softmax') %>%

compile(

optimizer = 'rmsprop',

loss = 'categorical_crossentropy',

metrics = c('accuracy')

)

In addition, our tool also supports users to design their own deep learning model for their own data in order to obtain more satisfactory results.

9.7 Model Results

Here, the model will train and test mass spectra data. In this process, the mass spectra of one file will be used in testing, the remaining data will be used in training in a for loop, which is similar to “leave-one-out” method. And then the classification results will be displayed including Confusion matrix, Heatmap, and ROC curve.


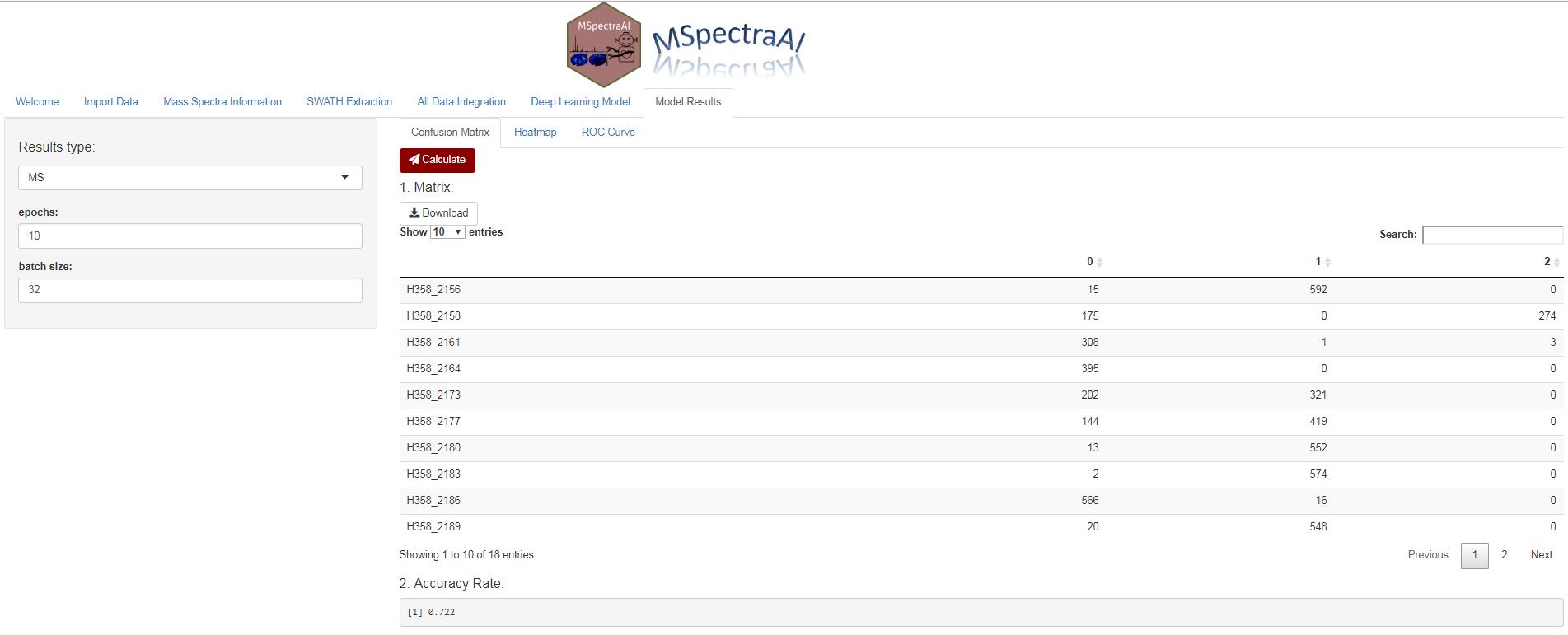


The parameter panel of this part is like this:


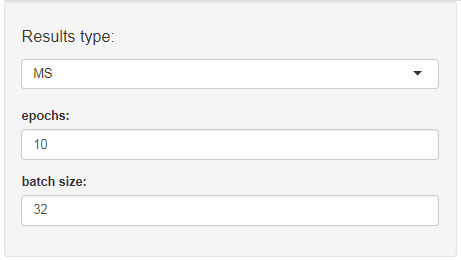


Results type: the results of MS1 or MS2 mass spectra data that users can choose to display.

epochs: number of epochs to train the model in fit function of keras package.

batch size: number of samples per gradient update in fit function of keras package.

Then click “Calculate” button to obtain the results.

For Confusion Matrix:


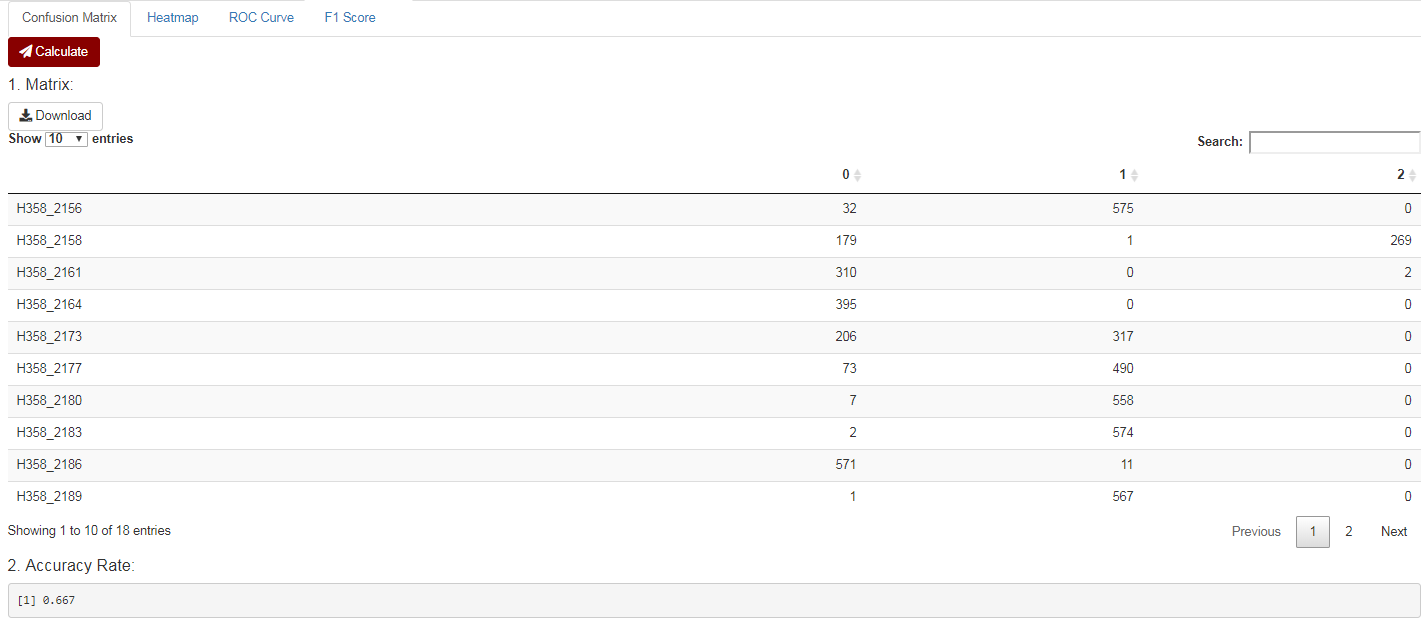


Here, the matrix contains predicted mass spectra labels for every sample, on which the final accuracy rate can be calculated based, for example, in “H358_2156” sample, there are total 607 MS1 spectra, and then 592 spectra are predicted as “1” label, whose rate (592/607 = 0.975) is above 0.5 (the default threshold), so this sample is classified as “1”. Repeatedly in this way, every sample can be predicted. If the predicted label is identical to that actual label, we think it is correct. For example data, 13 samples are predicted correctly, so the accuracy rate is 0.722 (13/18).

For Heatmap:


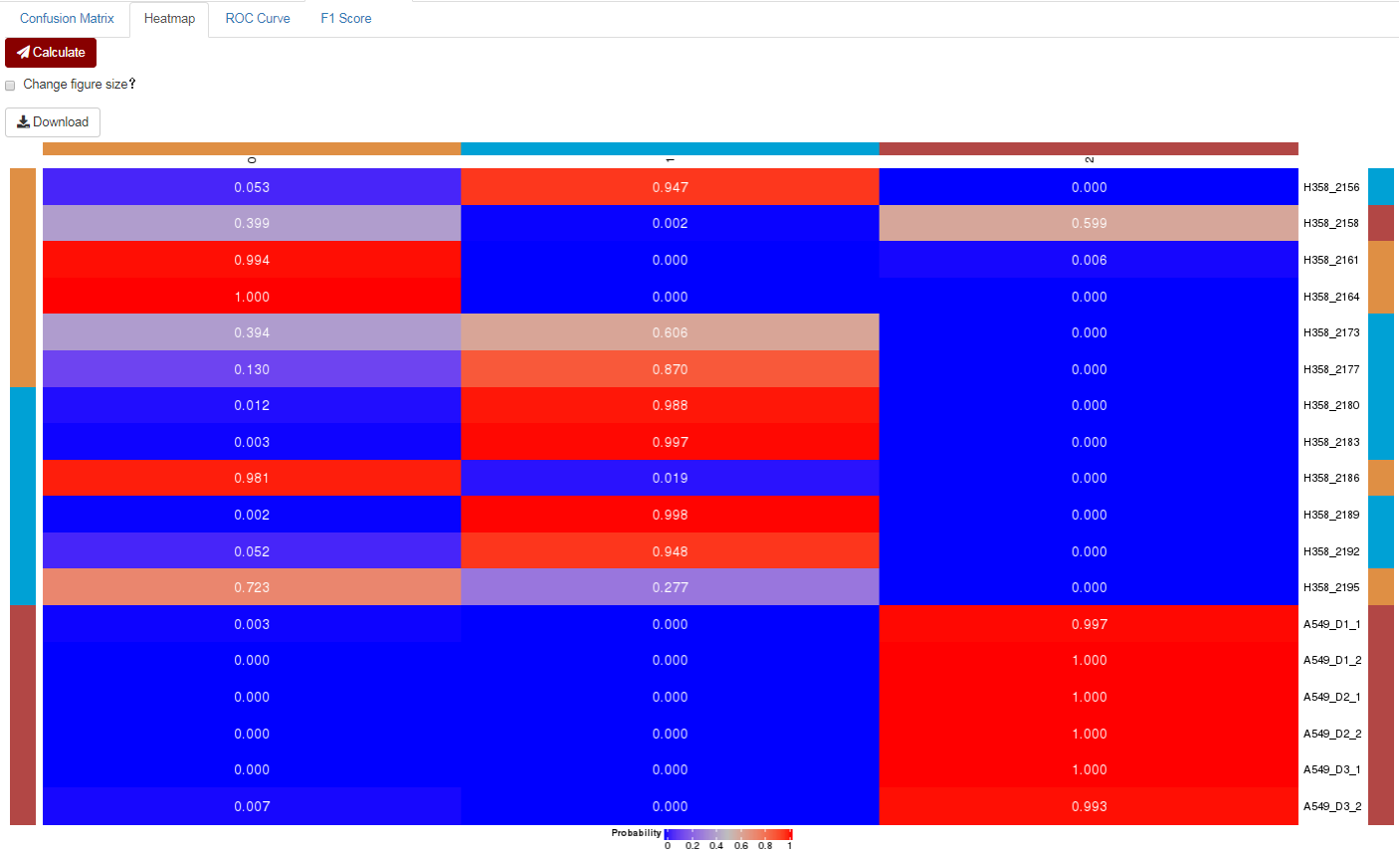


This is mainly visualised for confusion matrix result.

For ROC curve:


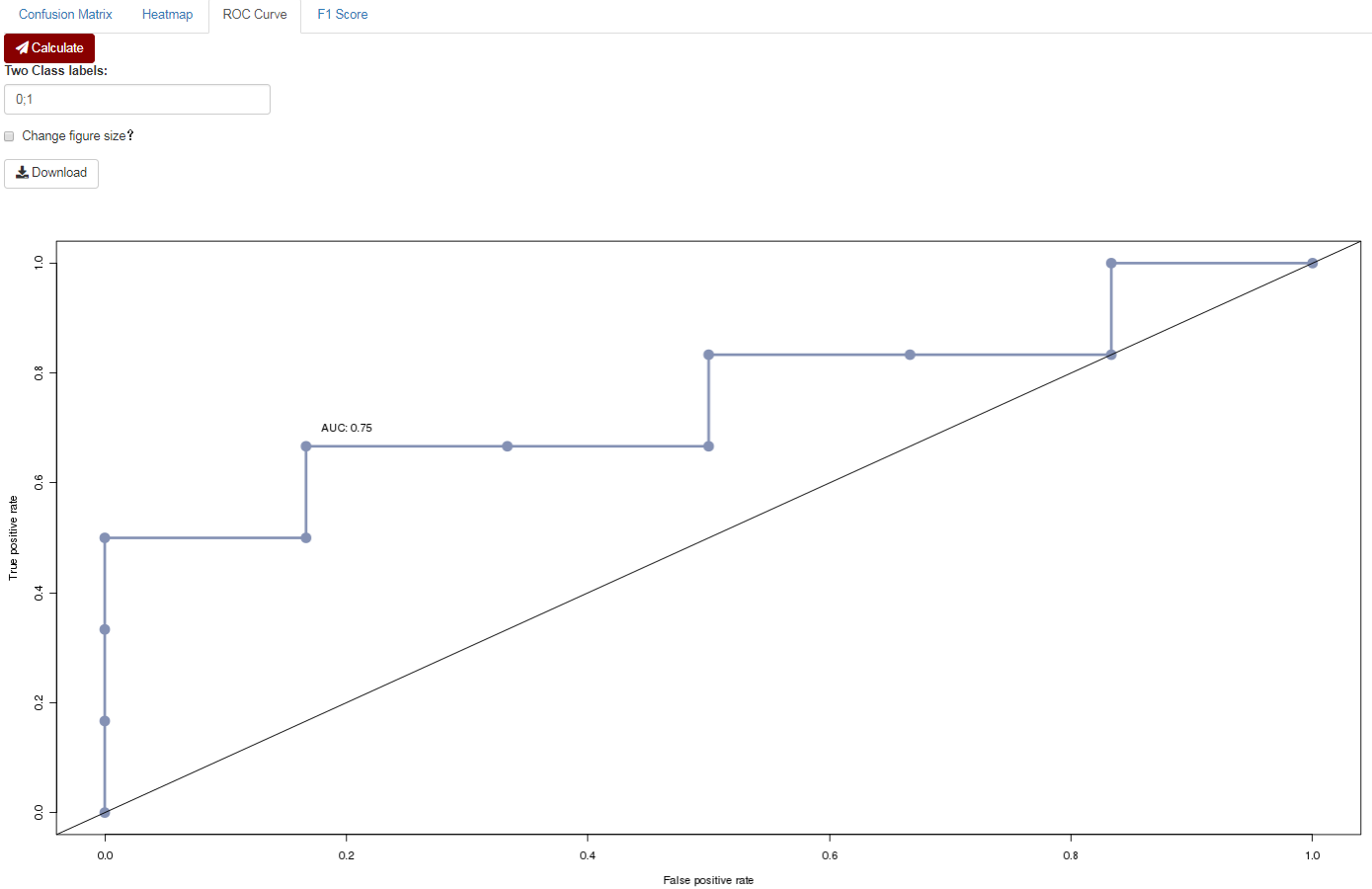


The receiver operating characteristic (ROC) curves and the area under the curve (AUC) are calculated using ROCR package [[9](#_ENREF_9)] for two-category samples which users can assign the class label in “Two Class Labels” box.

For F1 Score:


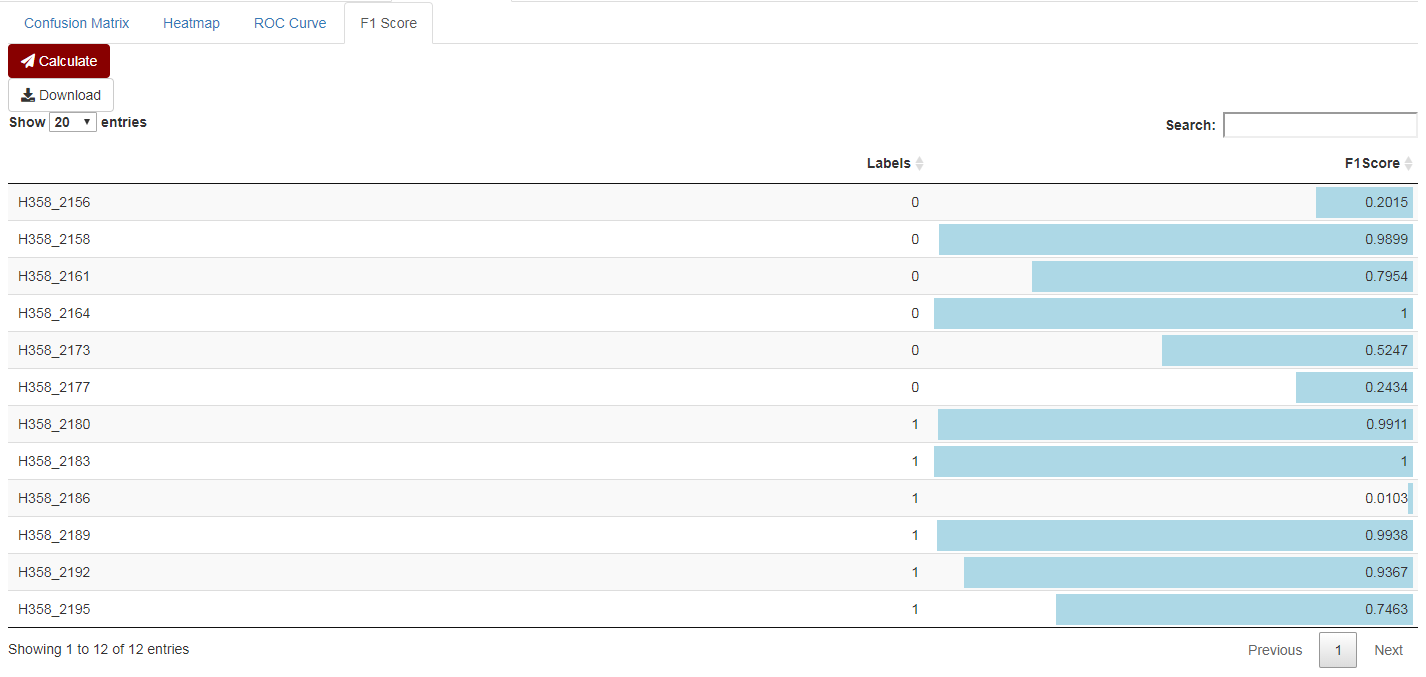


The F1 score is usually used as a measure of a test's accuracy for statistical analysis of binary classification. The class labels are same as those in “ROC Curve”. The colour bars in the “F1Score” column indicate the magnitude of these F1 scores.

**III. Supplementary Methods**

**1. Proteomic data analysis**

The raw MS data in every study (Table S1) were processed with MaxQuant software (Version 1.6.11.0) [[10](#_ENREF_10)] and searched against the Homo sapiens Uniprot database (20430 reviewed protein sequences, downloaded on 08-10-2019) supplemented with 246 frequently observed contaminants located in the file conf/contaminants.fasta in the MaxQuant software. The search parameters were kept similar or default as described in the corresponding published paper, whereas, the ‘Match between runs’ function was enabled, the ‘Label-free quantification’ was set to ‘LFQ’ with default parameters, and the false discovery rate (FDR) was set to 1% at both the peptide and the protein level using a target-decoy (forward-reverse) database searching strategy across all re-search process in this work. All proteome matrix data (proteinGroups.txt files generated by MaxQuant) can be downloaded from here:

<https://github.com/wangshisheng/MSpectraAI/tree/master/ProteomeData/>.

Afterwards, protein abundance matrix was calculated based on the LFQ intensity and the median value of each sample was used to normalize the intensity in order to alleviate the deviation of sample sizes and loading amounts in the MS analysis process. Then the intensities were log2-transformed and the datasets were filtered by removing those proteins with over 50% missing values. The remaining missing values were imputed with k-nearest-neighbour method (k=10) [[11](#_ENREF_11), [12](#_ENREF_12)] and those proteins with high coefficient of variation (above 30%) were also removed. Finally, the intact data of each study was processed by the following deep neural network model. All data analysis in this part was performed in R version 3.6.3 [[13](#_ENREF_13)].

**2. Deep neural networks to predict the proteome matrix data**

Herein, we first transposed the data obtained from the proteomic data analysis step (rows were samples, columns were proteins) and then applied z-score normalization to process these data. Next, we built a similar deep neural network model (Figure S2) to analyse the normalized data with the same leave-one-out cross prediction strategy as implemented in MSpectraAI and evaluated the performance based on four common criteria (Accuracy, Sensitivity, Precision, and F1 score). The deep neural network model was also implemented with keras package [[14](#_ENREF_14)] in R (Version 3.6.3).

**IV. References**

1. Löffler MW, Kowalewski DJ, Backert L, Bernhardt J, Adam P, Schuster H, Dengler F, Backes D, Kopp H-G, Beckert S: Mapping the HLA ligandome of Colorectal Cancer Reveals an Imprint of Malignant Cell Transformation. *Cancer research* 2018:canres. 1745.2017.

2. Jin J, Son M, Kim H, Kim H, Kong S-H, Kim HK, Kim Y, Han D: Comparative proteomic analysis of human malignant ascitic fluids for the development of gastric cancer biomarkers. *Clinical biochemistry* 2018, 56:55-61.

3. Wiredja DD, Ayati M, Mazhar S, Sangodkar J, Maxwell S, Schlatzer D, Narla G, Koyutürk M, Chance MR: Phosphoproteomics Profiling of Nonsmall Cell Lung Cancer Cells Treated with a Novel Phosphatase Activator. *Proteomics* 2017, 17(22):1700214.

4. Bohnenberger H, Kaderali L, Ströbel P, Yepes D, Plessmann U, Dharia NV, Yao S, Heydt C, Merkelbach‐Bruse S, Emmert A: Comparative proteomics reveals a diagnostic signature for pulmonary head‐and‐neck cancer metastasis. *EMBO molecular medicine* 2018, 10(9):e8428.

5. Zagorac I, Fernandez-Gaitero S, Penning R, Post H, Bueno MJ, Mouron S, Manso L, Morente MM, Alonso S, Serra V: In vivo phosphoproteomics reveals kinase activity profiles that predict treatment outcome in triple-negative breast cancer. *Nature communications* 2018, 9(1):3501.

6. Carnielli CM, Macedo CCS, De Rossi T, Granato DC, Rivera C, Domingues RR, Pauletti BA, Yokoo S, Heberle H, Busso-Lopes AF *et al*: Combining discovery and targeted proteomics reveals a prognostic signature in oral cancer. *Nature communications* 2018, 9(1):3598.

7. He L, Diedrich J, Chu Y-Y, Yates III JR: Extracting accurate precursor information for tandem mass spectra by RawConverter. *Anal Chem* 2015, 87(22):11361-11367.

8. Adusumilli R, Mallick P: Data conversion with ProteoWizard msConvert. In: *Proteomics.* Springer; 2017: 339-368.

9. Sing T, Sander O, Beerenwinkel N, Lengauer T: ROCR: visualizing classifier performance in R. *Bioinformatics* 2005, 21(20):3940-3941.

10. Cox J, Mann M: MaxQuant enables high peptide identification rates, individualized p.p.b.-range mass accuracies and proteome-wide protein quantification. *Nature biotechnology* 2008, 26(12):1367-1372.

11. Wang S, Li W, Hu L, Cheng J, Yang H, Liu Y: NAguideR: performing and prioritizing missing value imputations for consistent bottom-up proteomic analyses. *Nucleic acids research* 2020.

12. Troyanskaya O, Cantor M, Sherlock G, Brown P, Hastie T, Tibshirani R, Botstein D, Altman RB: Missing value estimation methods for DNA microarrays. *Bioinformatics* 2001, 17(6):520-525.

13. Ihaka R, Gentleman R: R: a language for data analysis and graphics. *Journal of computational and graphical statistics* 1996, 5(3):299-314.

14. Allaire J, Chollet F: keras: R Interface to’Keras’. *R package version* 2019, 2(4).
